# Genomic population structure of *Helicobacter pylori* Shanghai isolates and identification of genomic features uniquely linked with pathogenicity

**DOI:** 10.1080/21505594.2021.1920762

**Published:** 2021-04-27

**Authors:** Feng Yang, Jinghao Zhang, Su Wang, Zhaoyang Sun, Jun Zhou, Feng Li, Yue Liu, Li Ding, Yixin Liu, Wenjing Chi, Tao Liu, Yongqun He, Ping Xiang, Zhijun Bao, Michal A. Olszewski, Hu Zhao, Yanmei Zhang

**Affiliations:** a Department of Laboratory Medicine, Research Center on Aging and Medicine, Fudan University, Shanghai, China; b Department of Laboratory Medicine, Shanghai Key Laboratory of Clinical Geriatric Medicine, Shanghai, China; c Department of Laboratory Medicine, Huadong Hospital, Fudan University, Shanghai, China; d Department of Endoscopy, Huadong Hospital, Fudan University, Shanghai, China; e Unit for Laboratory Animal Medicine, Department of Microbiology and Immunology, And Center for Computational Medicine and Bioinformatics, University of Michigan Medical School, Ann Arbor, USA; f Department of Gastroenterology, Gerontology Institute of Shanghai, Huadong Hospital, Fudan University, Shanghai, China; g Division of Pulmonary and Critical Care Medicine, Department of Internal Medicine, University of Michigan and Research Service, VA Ann Arbor Healthcare System, Ann Arbor, USA

**Keywords:** *Helicobacter pylori*, gastric diseases, genomic features, pathogenicity, genomic island, crispr, population structure

## Abstract

Severe *Helicobacter pylori*-linked gastric disorders are especially prevalent in the East Asia region. The ability of *H. pylori* to cause different clinical outcomes is thought to be associated with unique sets of its genetic features. However, only few genetic features have been definitively linked to specific gastrointestinal pathologies. Genome heterogeneity of clinical *H. pylori* strains from patients with four different gastric disorders was studied to explore the population structure and molecular genomic features and their association with pathogenicity. Population analysis showed that 92.9% of the Shanghai *H. pylori* isolates were clustered in the East Asia group. Among 2,866 genes detected in all genomes, 1,146 genes formed the core genome, whereas 209 unique genes were detected in individual disease groups. The unique genes of peptic ulcer and gastric cancer groups represented the inorganic ion transport and metabolism function gene clusters. Sixteen virulence genes were detected with statistically different detection rates among the four disease groups. Furthermore, 127 clustered regularly interspaced short palindromic repeats were found with significantly different rates in the four disease groups. A total of 337 putative genomic islands were identified, and three genomic islands were individually found in more than 10% of strains. The genomic islands included several metabolism-associated genes and many genes with unknown function. In total, 88 sequence types were detected among the 112 Shanghai *H. pylori* isolates. Our study provides an essential milestone in the mapping of specific genomic features and their functions to identify factors needed to induce specific gastric disorders in *H. pylori.*

## Introduction

*Helicobacter pylori* infection is an important risk factor for severe gastritis-associated disorders, including peptic ulcer, gastric mucosa-associated lymphoid tissue lymphoma, and gastric cancer [1]. More than 40% of the world’s human population is persistently colonized with *H. pylori*, ranging from 35% in developed countries to over 50% in developing countries [2]. However, the clinical outcomes of infected patients vary significantly, and 10–15% of carriers develop *H. pylori* associated diseases [3]. Recent studies have suggested that pronounced *H. pylori* genome variability contributes to the non-uniform pathogenicity of *H. pylori* gastric colonization in different individuals [4,5].

Metagenomics studies have revealed the existence of geographically localized population structures in *H. pylori* isolates [6]. The population structure of *H. pylori* was investigated by analyzing the sequences of seven conserved genes (*atpA, efp, mutY, ppa, trpC, ureI*, and *yphC*). The structure is currently divided into seven population types based on geographical associations with the unique genetic features: hp-Europe, hp-EastAsia, hp-Africa1, hp-Africa2, hpAsia2, hp-NEAfrica, and hp-Sahul. The hp-EastAsia type is further divided into the hp-EAsia, hp-Amerind, and hp-Maori subpopulations. The hp-Africa1 type comprises the hp-WAfrica and hp-SAfrica subpopulations [7]. The various population structures of *H. pylori* are thought to reflect the pathway of the spread of these isolates related to historical migrations and resettlements of certain human populations and the varied distributions could be responsible for regional differences concerning the clinical consequences of gastric colonization by *H. pylori* [8,9].

The pathogenic mechanisms of *H. pylori* are complex. The most important of these include the expression of specific virulence genes. The most recognized is the cytotoxin-associated gene pathogenicity island (*cag*PAI), which encodes the CagA protein and type IV secretion system (T4SS) [10]. Other genes implicated in *H. pylori* adaptation and survival include genes encoding outer membrane proteins (OMPs) and flagellar genes [11,12]. Genes with variable genotypes or structures in specific isolates, such as the *vacA* gene, are also related to pathogenicity [13]. However, the degree to which these virulence factors and their variants are associated with specific clinical outcomes remains poorly understood.

Genomic islands (GIs) in prokaryotes are discrete inserted DNA segments acquired by horizontal transfer. Many GIs are mobile and can harbor genes that influence pathogenicity [14], antibiotic resistance [15], metabolism [16], and even bacteria-virus coexistence [17]. However, other than *cag*PAI, no other GIs have been studied in *H. pylori* clinical isolates. These deserve further exploration in the context of specific gastric pathologies.

In addition to protein-coding genomic regions, the role of non-coding genome regions in microbial virulence has been increasingly recognized. Clustered regularly interspaced short palindromic repeats (CRISPRs) consist of two components: spacers and direct repeats (DRs). CRISPR regions are detected in approximately 40% of bacteria and 90% of archaea. CRISPR-Cas (CRISPR-associated proteins) systems serve as prokaryotic immune systems that provide resistance against viruses (phages) and potentially harmful foreign plasmids and transposons. CRISPR interference could influence the acquisition of virulence during bacterial infection [18]. There are limited reports that CRISPR interference could prevent the emergence of virulence in certain bacteria, and the strong selective pressure for virulence or antibiotic resistance may also influence CRISPR emergence. Other evidence suggests that the CRISPR/Cas system and pathogenic associated genes influence each other and there might be certain CRISPR/virulence types [19]. However, understanding of CRISPRs in *H. pylori* and their possible virulence function is limited, especially in the context of particular gastrointestinal diseases.

The associations between prevalent genotypes with particular virulence attributes and geographical distribution have been demonstrated in clinical bacterial isolates [20]. Multi-locus sequence typing (MLST) was proposed as one of the most effective tools to investigate the prevalence and genetic diversity of pathogenic bacteria in a certain area [21]. However, the predominant sequence types (STs) of *H. pylori* in Shanghai, China, await detailed MLST evaluation.

Whole-genome analysis enables the rapid assessment of the genetic diversity in *H. pylori* isolates and its relationship with clinical outcomes resulting from *H. pylori* colonization. This information is important in identifying the pathogen-induced mechanisms of *H. pylori* that promote the development of specific gastrointestinal pathologies. In this study, we explored the genome characteristics of *H. pylori* strains from individuals with different clinical outcomes, including chronic superficial gastritis (CSG), chronic atrophic gastritis (CAG), peptic ulcer (GU), and gastric cancer (GC). We identified the specific genetic features of *H. pylori* and genetic heterogeneity related to specific clinical outcomes and, where possible, determined the functions of these genes. The data broaden insight of the diversity among *H. pylori* and their linkage to specific gastric diseases.

## Materials and methods

### H. pylori isolates

One hundred and twelve *H. pylori* isolates were isolated from gastric biopsies of patients with gastric diseases who resided in Shanghai (*H. pylori-*Shi isolates) (Supplemental Table S1). Based on the diagnosis, the isolates were divided into four groups: CSG (n = 19), CAG (n = 66), GU (n = 26), and GC (n = 1). With the consent of patients who underwent gastric biopsy, two pieces of tissue collected from the antrum 2–3 cm front of the pylorus were used for bacterial culture and pathological examination. Pathological examinations of the gastric biopsies were performed by clinical pathologists at Huadong Hospital in Shanghai. The study was approved by the hospitals’ Ethics Committee for Human Studies, with written informed consent from all subjects (Ethics Approval Number: 2020K080). To analyze the phylogeny and association between sequence features and clinical outcomes, genome sequences of 10 reference strains isolated from patients with GC were downloaded from GenBank. Detailed information is provided in Supplemental Table S2.

### Genomic DNA extraction

*H. pylori* isolates were plated on Columbia agar (OXOID, Thermo Fisher Scientific Inc., Waltham, MA, USA) medium containing 8% sterile defibrinated sheep blood under microaerophilic conditions at 35°C for 3–5 days. After three subcultures, the genomic DNA was extracted using the cetyltrimethylammonium bromide method [22].

### Genome sequencing and data assembly

Genome sequencing was performed using the MiSeq platform (Illumina, San Diego, CA, USA) by generating paired-end libraries. Genomic DNA libraries for each isolate were prepared using the TruSeq DNA Sample Preparation Kit (Illumina). Adapter contamination was removed by AdapterRemoval v2 [23] and the reads were filtered by SOAPec v2 [24]. The filtered reads were assembled into contigs and scaffolds using A5-miseq v20160825 [25]. The sequenced genomes of the 112 *H. pylori-*Shi isolates were submitted to the National Center for Biotechnology Information (NCBI).

### Analysis of population structure

To assign the 112 newly sequenced *H. pylori*-Shi isolates to the previously described population types, we compared the sequences of 7 housekeeping genes (*atpA, efp, mutY, ppa, trpC, ureI*, and *yphC*, which are conserved genes related to basic life activities including ATP synthesis, transcription, and used for MLST traditionally) in each isolate with 120 corresponding comparative sequences of *H. pylori* exported from the PubMLST database (https://pubmlst.org). These comparative sequences were obtained from a broad range of geographic locations and contained 10 population types (Supplemental Table S 3). Nucleotide sequences of the seven concatenated genes were aligned using the ClustalW algorithm within MEGA4. Phylogenetic relationships were constructed using MEGA4 with the Kimura 2-parameter model of nucleotide substitution and neighbor-joining clustering.

### Core genome analysis

The predicted protein sequences of all 122 *H. pylori* strains were compared to identify orthologous gene clusters using OrthoMCL v2.0.8. Sequences of a minimum of 50 amino acids were considered for further ortholog analysis. The length of sequence alignment and E-value were set at 70% and 1e-10, respectively. The identified clusters were analyzed to calculate the core genome consisting of genes shared by all 122 strains and the clinical outcome-specific genes. The clusters of orthologous groups of proteins (COG) database (http://eggnogdb.embl.de/#/app/home/) was used for functional classifications. Genes that failed to align with a suitable “hit” within the database were categorized as hypothetical genes [26].

### Gene prediction and annotation

Genome function element prediction included the prediction of coding genes (CDSs), non-coding RNAs, and CRISPRs. Open reading frames (ORFs) were predicted by Glimmer v3.02 [27], tRNA genes were predicted using tRNAscan-SE v1.3.1 (http://lowelab.ucsc.edu/tRNAscan-SE/), and rRNA genes were predicted by Barrnap v0.9 [28,29]. CRISPRs were identified using CRISPRfinder v20170509 (http://crispr.i2bc.paris-saclay.fr/Server/). Functional annotation of ORFs was conducted using BLAST2GO v1.0 (https://www.blast2go.com/) based on the Gene Ontology database (http://www.geneontology.org/) and the Kyoto Encyclopedia of Genes and Genomes (KEGG) annotation using the KEGG automatic annotation server (KASS v2.1, https://www.genome.jp/tools/kaas/) with the KEGG database (http://www.genome.jp/kegg/). The Virulence Factors of Pathogenic Bacteria (VFDB) database (http://www.mgc.ac.cn/VFs/main.htm) was used to retrieve pathogenicity-associated genes. The Victors database (http://www.phidias.us/victors) was also used to detect virulence genes [30]. GIs were detected by IslandViewer 4 (http://www.pathogenomics.sfu.ca/islandviewer/upload/) [31].

### MLST analysis

The sequences of seven standard MLST genes (*atpA, efp, mutY, ppa, trpC, ureI*, and *yphC*) from 112 *H. pylori*-Shi isolates were submitted to the *H. pylori* MLST database (http://pubmlst.org/Helicobacter) for allele and sequence type identification. If there was not an exact match for the seven alleles in the database, they were submitted to the curator for definition.

### Statistical analyses

Statistical analyses were performed using Stata/SE 14.0 (Stata Corp, College Station, TX, USA) for Mac. Differences between the groups were compared using Chi-square or Pearson Chi-square tests, Fisher’s exact method, or Kruskal-Wallis analysis according to the data type and the number of comparisons. Statistical significance was defined as *P* <0.05. All *P*-values were two-sided. When the results of Pearson Chi-square tests showed a statistical difference, the intra-group pairwise comparison was adjusted using the Bonferroni formula.

## Results

### General genomic features and population structure of H. pylori-Shi isolates

The draft genome sequences of 112 *H. pylori*-Shi revealed chromosome sizes ranging from 1.52 to 1.69 Mb. The genomes had a low average G + C content of 38.7% with 1,511 to 1,624 genes per genome. All the sequenced genomes harbored 36 tRNA genes and two rRNA genes. The genome accessions of NCBI and detailed genomic features grouped by the clinical diagnosis of the patients were listed in Supplemental Table S4.

**Figure 1.**
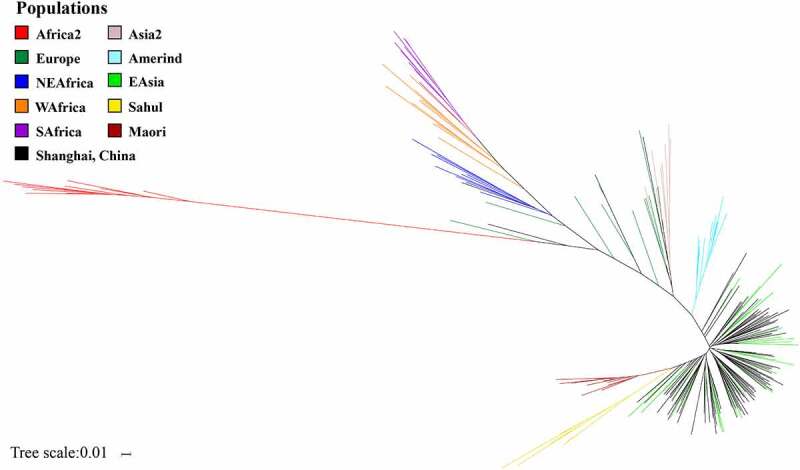
Contemporary population structure of Shanghai *H. pylori* isolates (*H. pylori*-Shi) largely overlaps with that of the entire East Asia (EAsia) cluster with minimal infusion of broader Eurasian clusters

To further validate that the sequencing data were representative and with sufficient breadth to represent the *H. pylori* genetic diversity in the East Asian region, we performed population structure analysis by comparing the seven MLST housekeeping genes in previously deposited *H. pylori* genome sequences from different geographic regions (*N*= 120) relative to those in our *H. pylori*-Shi isolates (*N*= 112) (Figure 1). Nearly all (104/112, 92.9%) of the *H. pylori-*Shi isolate clusters overlapped with the EAsia isolate group, covering the full spectrum of variants previously reported within this region. Additionally, 6.3% (7/112) of the isolates clustered with European variants and 0.9% (1/112) clustered within other regions of Asia. In contrast, the *H. pylori-*Shi isolates were distinctly separated from other regional clusters (Maori, Amerind, Africa2, South Africa, West Africa, Asia2, and Sahul). Thus, in terms of genetic makeup and variation, the *H. pylori-*Shi isolates overlapped well with those previously reported in the East Asian region with some additional minor admixture from broader Eurasian *H. pylori* genetic clusters.

The population structure of 112 *H. pylori*-Shi was analyzed by comparing the sequences of seven housekeeping genes with other 120 comparative sequences belonging to the 10 major population clusters. Most *H. pylori*-Shi isolates are clustered within the EAsia group.

### Core genome and clinical outcome-specific genes of H. pylori strains

**Figure 2.**
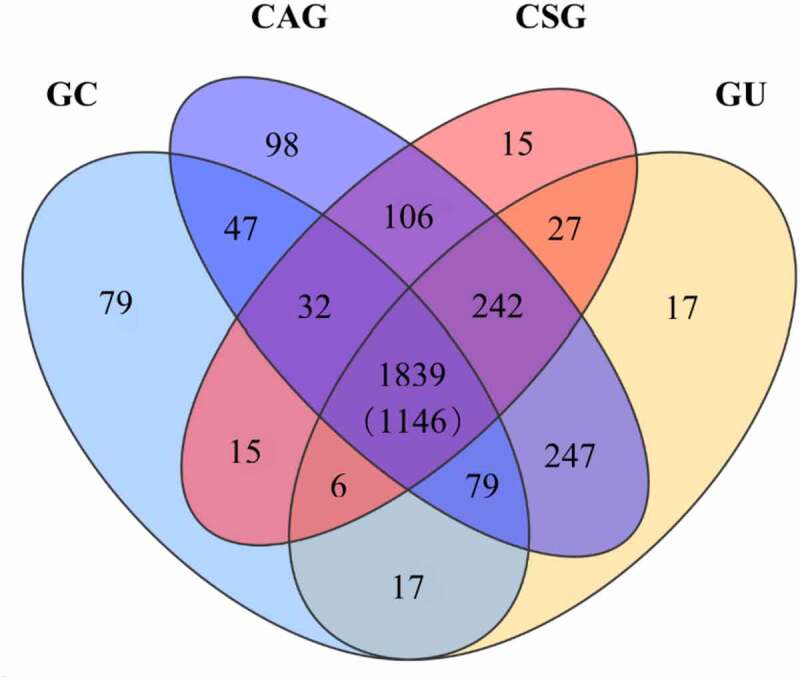
Many identified *H. pylori* genes exclusive of the *H. pylori* Core Genome include subsets of genes that appear to be linked with specific pathological outcomes

**Figure 3.**
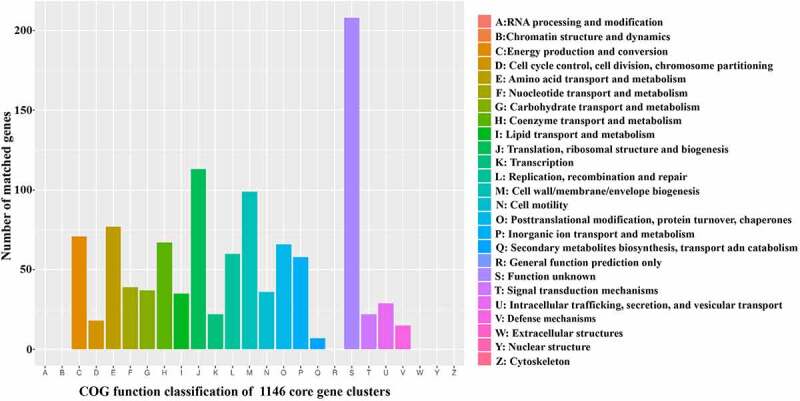
Functional classification of core genes in 122 *H. pylori* strains reveals predominant basic structure and metabolism genes as well as a group of genes with unknown functions

To further explore the genetic diversity *of H. pylori* in relation to the clinical outcomes, we sought to define the core genetic content and genes related to certain clinical outcomes. The genes that shared orthologs in the genomes of all strains were classified as the core genome pool. The genomes of the 112 *H. pylori-*Shi isolates and 10 reference strains of gastric cancer were analyzed to define which genes constituted the core genome pool and which could be categorized as clinical outcome-related genes. Because different genomes possessed a unique combination of genes, the total number of distinct genes found in all genomes was 2,866. Among these genes, 1,146 formed the core genome pool that was present in all genomes. The remaining 1720 genes occurred in a subset of strains, including 209 clinical outcome-specific genes (Figure 2). Among the 1,146 core genes, 1,079 could be assigned to various COG functional classes (Figure 3). A high proportion of genes had an unknown function (class S, Supplemental Table S 5). The domain categories were related to basic life activities, including J (translation, ribosomal structure, and biogenesis), E (amino acid transport and metabolism), and M (cell membrane/envelope biogenesis) functional classes. Genes predicted to be related to inorganic ion transport and metabolism (class P) also accounted for a large proportion. Among the genes uniquely found in each disease group, eight of 15 CSG specific genes could be assigned to COG categories that were enriched in the function of DNA replication, recombination, and repair (Table 1). Thirty-five of 98 CAG specific genes could be assigned to COG whose distribution was dispersive in each function category. Three of 17 GU specific genes could be assigned to COG with unknown function. Fifty of 79 GC specific genes could be assigned to COG, among which the dominant known function was inorganic ion transport and metabolism. Notably, the GC/GU specific genes were also enriched for inorganic ion metabolism.

**Table 1.** The functions of the clinical outcome specific gene clusters

Different groups	Specific genes	Annotated genes	COG function classifications																
			C	D	E	F	G	H	I	J	L	M	N	O	P	S	T	U	V
CSG	15	8	1	1	1	-	-	-	-	-	3	-	-	-	-	2	-	-	-
CAG	98	35	3	2	-	2	-	3	1	-	3	4	3	2	4	4	-	2	2
GU	17	3	-	-	-	-	-	-	-	-	-	-	-	-	-	3	-	-	-
GC	79	50	2	-	1	-	1	-	2	3	5	5	2	4	6	16	1	1	1
GC+GU	17	2	-	-	-	-	-	-	-	-	-	-	-	-	2	-	-	-	-

Note that the GC/GU specific genes are enriched for class P (inorganic ion transport and metabolism), consistent with the role of microbial ion transport pathways in driving GC/GU pathological processes. The annotations of each COG functional classes are as per Figure 3, (*leaving out classes without a single hit*).

A total of 2,866 genes were detected from 122 *H. pylori* strains. Of these, 1,146 genes constituted the core genome shared by all isolates. There were 1,839 genes present in the isolates of the four disease groups, but not necessarily in all the isolates. The overlapping areas contain genes that are detected in multiple corresponding clinical outcome groups. Abbreviations: GC, gastric carcinoma; GU, peptic ulcer; CAG, chronic atrophic gastritis; CSG, chronic superficial gastritis.

The functional classifications of 1,146 core genes were explored in the COG database. The X-axis denotes the functional category. The Y-axis represents the number of genes in each category. The detailed functions are represented in the right panel. Most core genes are involved in basic bacterial structure and metabolism. However, there was also a high proportion of functionally unknown genes, which could contain genes functioning in *H. pylori* pathogenesis pathways.

### Differential distribution of virulence-associated genes in specific clinical outcome groups

To investigate whether the occurrence of specific virulence genes *H. pylori* carried was related to specific clinical outcomes, the status of 115 virulence-associated genes in four groups was analyzed against the VFDB database, including 26 *cag* pathogenicity island (*cag*PAI) genes and 89 others (Supplemental Table S 6). Most (113 of 122) *H. pylori* strains carried *cag*PAI. Remarkably, among the 113 strains presenting *cag*PAI, complete *cag*PAI was detected in only 12 (10.6%) strains, which possessed all 26 major genes. The remaining 101 (89.4%) carried partial *cag*PAI. In addition, 66.7% (8/12) of strains carrying intact *cag*PAI were detected in patients with CAG, 25.0% (3/12) in those with CSG, and 8.3% (1/12) in those with GU. There was no statistical difference in *cag*PAI detection rates among the four groups. The other 89 virulence-associated genes, including OMP genes (*hopS, hopT*, etc.), flagellar protein genes (*fliL, flgG*, etc.), urease accessory protein genes (*ureA, ureB*, etc.), genes encodeing enzymes (*futC, futB*, etc.), and vacuolating cytotoxin gene (*vacA*) were also identified. Notably, 59.6% (53/89) genes were detected in all 122 *H. pylori* strains. In addition, 20 other virulence genes were detected by the Victors database. Of these, 18 were present in all 122 strains. The other two genes, *katA* (codes for catalase) and *homB* (codes for outer membrane protein), displayed apparently distinct distribution among the four groups, but without statistical significance (Supplemental Table S 7).

The association of 115 virulence-associated genes with clinical outcomes was further investigated. Sixteen virulence genes with statistically different detection rates among the four groups included four *cag*PAI genes and 12 other virulence-associated genes (Table 2). The GC group was consistently showing either significantly higher or lower positive detection rates among these 16 genes compared with all other clinical groups. The four *cag*PAI genes (*cagP, cagH, cagY*, and *cag5*) performed corresponding functions in the T4SS. The cagP encoded by *cagP* plays a role in *H. pylori* adherence to gastric epithelial cells, cagH encoded by *cagH* is involved in pilus biogenesis, cagY encoded by *cagY* functions as an immune-sensitive regulator of T4SS, and virD4 encoded by *cag5* acts as an adapter protein for the transfer of CagA protein. The other 12 genes were *flgM, flgR, flhB, flhB2, fliF, fliM, fliQ, babA/hopS, futA, futB, galE*, and *ureB*. The *flgM, flgR, flhB, flhB2, fliF, fliM*, and *fliQ* genes are all flagellum genes. They are essential for the structure and function of flagella, while the presence of these flagellar genes was not consistently associated with the severity of clinical outcomes. The *babA/hopS* codes for adhesin BabA, which mediates adhesion to the host cell. The *futA* and *futB* genes code for fucosyltransferases (FutA and FutB, respectively), which are linked with the structure of lipopolysaccharide (LPS). The *galE* gene is also related to the synthesis of LPS in *H. pylori*. Finally, the *ureB* gene codes for urease beta subunit (UreB), which could help counterbalance the acidic environment in the gastric mucosa to improve survival of *H. pylori*.

**Table 2.** Sixteen virulence genes demonstrate that statistically different frequency of detection among the clinical outcome groups

Genes	Number and % gene positive isolates in each clinical category				*P*
	CAG (n = 66)	CSG (n = 19)	GU (n = 26)	GC (n = 11)	
*cagH*	61 (92.4%)	18 (94.7%)	24 (92.3%)	6 (54.5%)	0.008
*cagP*	9 (13.6%)	3 (15.8%)	4 (15.4%)	6 (54.5%)	0.027
*virB10/cagY*	44 (66.7%)	11 (57.9%)	17 (65.4%)	1 (9.1%)	0.004
*virD4/cag5*	61 (92.4%)	19 (100.0%)	24 (92.3%)	6 (54.5%)	0.003
*flgM*	66 (100.0%)	19 (100.0%)	26 (100.0%)	4 (36.4%)	0.000
*flgR*	66 (100.0%)	17 (89.5%)	26 (100.0%)	8 (72.7%)	0.000
*flhB*	66 (100.0%)	19 (100.0%)	26 (100.0%)	8 (72.7%)	0.001
*flhB2*	66 (100.0%)	17 (89.5%)	26 (100.0%)	11 (100.0%)	0.031
*fliF*	66 (100.0%)	19 (100.0%)	26 (100.0%)	7 (63.6%)	0.000
*fliM*	66 (100.0%)	19 (100.0%)	26 (100.0%)	9 (81.8%)	0.007
*fliQ*	66 (100.0%)	19 (100.0%)	26 (100.0%)	1 (9.1%)	0.000
*babA/hopS*	8 (12.1%)	4 (21.1%)	7 (26.9%)	9 (81.8%)	0.000
*futA*	8 (12.1%)	1 (5.3%)	6 (23.1%)	9 (81.8%)	0.000
*futB*	8 (12.1%)	1 (5.3%)	6 (23.1%)	9 (81.8%)	0.000
*galE*	66 (100.0%)	18 (94.7%)	25 (96.2%)	9 (81.8%)	0.015
*ureB*	66 (100.0%)	19 (100.0%)	26 (100.0%)	9 (81.8%)	0.007

Among known virulence-associated genes registered in VFDB database, 16 show significantly different distribution in four clinical outcome groups (*Note: These and the additional virulence genes that show no significant difference in frequencies among four clinical groups are all shown in Supplemental Table S5*).

***Copy numbers of the 115 virulence-associated genes in H. pylori strains of specific clinical outcome groups***

**Figure 4.**
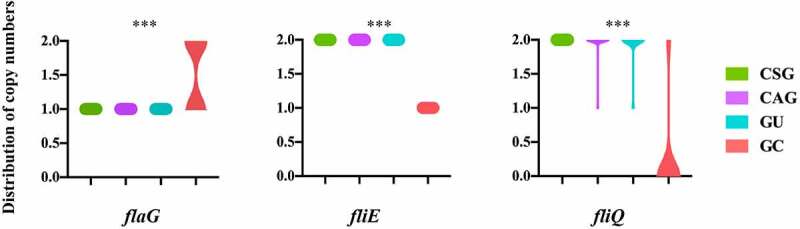
Three virulence-associated genes with significant different copy numbers among four clinical outcome groups

To further explore the association between the copy numbers of the 115 virulence-associated genes and clinical outcomes, the variation in copy numbers of these genes in 122 *H. pylori* was analyzed (Supplemental Figure S1). Four copies were only detected in gene *pseB*, which participates in the modification of flagellin in one strain. Three copies were detected in *flgG*, which encodes flagellar protein and *babB/hopT*, which encodes the OMP BabB as an adhesin in 121 and 14 strains, respectively. Three genes (*fliQ, flaG*, and *fliE*) displayed significantly different numbers of copies in isolates from different groups (Figure 4). Notably, GC was always notable in terms of these three-copy numbers, which tended to be over- or under-represented, relative to all the remainder. As shown above, the presence of *fliQ* in the GC group was significantly lower than that in the other groups, and its copy number also varied significantly in different groups. While there was no significant difference in the presence of *flaG* and *fliE* among the different clinical outcome groups (Supplemental Table S 6), their average copies were statistically lower in the GC groups. Thus, the GC group was associated with the greatest dispersion in the number of copies of the three virulence-associated genes.

The distribution of copy numbers of three virulence-associated genes significantly related with clinical outcomes (excluding the genes presenting either one copy or absence in different isolates). Note that the presence of GC was associated with the greatest dispersion in a number of copies of these genes; ***P< 0.001.

### Putative GIs detected in various clinical outcome groups

**Figure 5.**
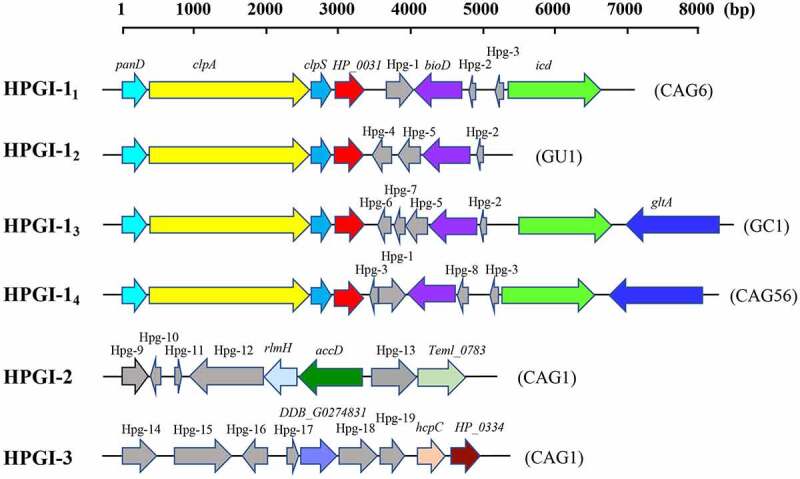
Structure analysis of three most common putative GIs in *H. pylori* strains identifies known genes and multiple unknown hypothetical protein genes that could be related to pathogenicity

GIs contribute to lateral gene transfer and bacterial evolution. Thus, we analyzed the presence of GIs in the 122 *H. pylori* strains. A total of 337 putative GIs were identified. They ranged in length from 1,875 to 71,269 bp. Almost all (120/122, 98.4%) of the strains possessed putative GIs. The frequency of GI was high in all four groups, with no significant difference (Supplemental Table S8). The top three most frequent GIs were HPGI-1 (45.9%, 56/122), HPGI-2 (12.3%, 15/122), and HPGI-3 (10.7%, 13/122) (Figure 5). The putative HPGI-1 was predicted to contain a variable number of CDSs involved in the aerobic oxidation of glucose and other nutrient metabolism, including several constantly present genes, such as *clpS* encoding chaperone-proteases adaptor protein, and some randomly occurring genes, such as *gltA*, which encodes citrate synthase. Four predominant genetic compositions of HPGI-1 (HPGI-1_1_-HPGI-1_4_) were shown in Figure 5. HPGI-1_1_ was detected in 15 strains, HPGI-1_2_ in four, HPGI-1_3_ in one, and HPGI-1_4_ in five. The putative HPGI-2 contained genes that included *rlmH* encoding ribosomal RNA large subunit methyltransferase and *Teml_0783* encoding competence damage-inducible protein A (CinA)-like protein (Figure 5, HPGI-2). HPGI-3 contained genes that included *HP_0334* coding Holliday junction resolvase-like protein, which is involved in homologous recombination (Figure 5, HPGI-3). Remarkably, the most frequently occurring putative GIs in *H. pylori* harbored many more genes with unknown function. These genes might encode hypothetical proteins or might be classified as genomic DNA. This warrants further attention. The presence of each GI did not differ among the four clinical outcome groups (*P*= 0.151, 0.191, and 0.337 for HPGI-1, HPGI-2, and HPGI-3, respectively).

Genetic maps of the top three predominant putative GIs. The same gene is shown in the same color. Genes with unknown function are shown in gray and marked as Hpg-1 through Hpg-19. The sequence of HPGI-1_1-4_ can be accessed in NCBI by accession number JAEQAQ000000000 (contig5, 92,805–99278), JAEPXO000000000 (contig2, 200,442–205283), JAEPWO000000000 (contig3, 93,861–101918), and JAEPYS000000000 (contig4, 92,585–100480), respectively. HPGI-2 by JAEQAV000000000 (contig9, 22,376–27014) and HPGI-3 by JAEQAV000000000 (contig2, 116,684–121408). The three most frequently occurring putative GIs in *H. pylori* possess multiple genes with unknown function. Abbreviations: Hpg, hypothetical protein gene; GI, genomic island.

### CRISPRs detected in various clinical outcome groups

**Figure 6.**
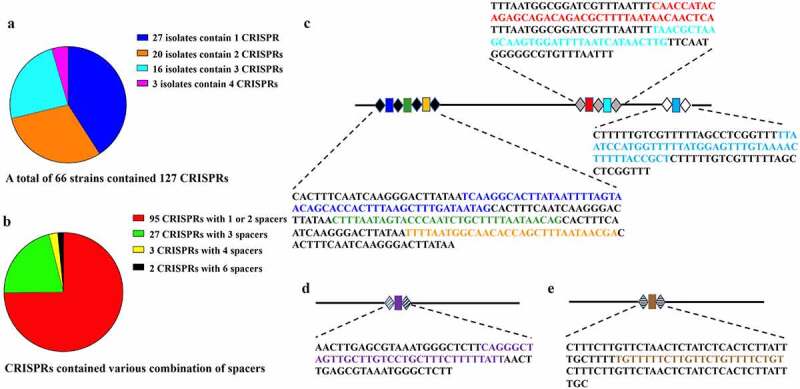
Distribution and constitution of CRISPRs and the three most predominant and previously unknown CRISPRs in *H. pylori* strains

Finally, given the unknown role of *H. pylori* CRISPRs in gastrointestinal pathogenesis, we studied the occurrence of CRISPRs in strains from the four groups. CRISPRs were detected in 45.5% (5/11) of GC strains, 80.8% (21/26) of GU strains, 48.5% (32/66) of CAG strains, and 42.1% (8/19) of CSG strains. The CRISPIR frequencies differed significantly among the four groups (*P*= 0.021; Supplemental Table S 9). Further intra-group statistical comparisons showed that the presence in the GU group (80.8%) was significantly higher than that in the CSG and CAG groups. In total, 66 *H. pylori* isolates contained 127 CRISPR loci. The length and spacer content in *H. pylori* CRISPRs varied distinctly (Figure 6(a,b)). A total of 79 distinct types of direct repeat (DR) were detected with lengths of 23–50 bp. Of these, 17 types of DR were detected more than once. Notably, 15 of the 17 repetitive DRs always corresponded to certain specific spacers. The most common DRs in *H. pylori* CRISPRs were found in nine, eight, and six isolates, respectively (Figure 6(c–e)). Interestingly, the CRISPRs were either precisely the same or entirely different among different isolates. The sequences of CRISPRs within the same isolate were not identical. No Cas genes were identified in any *H. pylori* isolate. Thus, although CRISPRs were significantly increased in GU *H. pylori* isolates, they did not arise through gene duplication and variation but represented highly conserved DNA fragments that presumably incorporated into the genome of the specific *H. pylori* isolates.

A total of 66 strains contained 127 CRISPRs with various compositions (A, B). The three most predominant CRISPRs or CRISPR combination (C, D, and E) were detected. No association with clinical outcome was found. The structural analysis of the most common CRISPRs (or CPISPR combination) defined the direct repeats (DRs, shown as black, gray, or white diamonds in each CRISPR) and eight unique spacers (shown as colored rectangles). The base sequences are shown near the CRISPR arrays. DRs are denoted by black uppercase characters. The spacers in the colored characters correspond with the colors of the respective rectangles. These three CRISPR/CRISPR combinations are reported for the first time.

### MLST analysis of the H. pylori-Shi isolates

MLST classified the 112 *H. pylori*-Shi isolates into 88 STs (Supplemental Table S10). The predominant STs were ST3274 and ST3327, which were detected in 13.4% and 6.3% of the isolates, respectively. There was no statistical difference in the distribution of the STs in the different groups (*P*= 0.316 and 0.667 for ST3274 and ST3327, respectively). Thus, the STs of *H. pylori*-Shi isolates were dispersive and were not associated with disease background.

## Discussion

The ability of *H. pylori* to cause different clinical problems and to establish lifelong gastrointestinal infections is thought to be associated with unique sets of genetic features [32,33]. However, only few of these features have been conclusively associated with specific gastrointestinal pathologies. In this study, the genetic features of 122 *H. pylori* strains from patients with four major gastric diseases (GSC, CAG, GU, and GC) were analyzed to explore possible links between specific genes and these gastrointestinal disorders’ epidemiology. We have found that: 1) genetic population structure of *H. pylori*-Shi isolates overlaid largely with that of the entire East Asia region, which largely also shares similar epidemiological landscape of the *H. pylori*-linked gastric problems; 2) the frequencies of 16 virulence-associated genes, including *cagP, cagH, flgM*, and the differences in the copy numbers of three genes (*fliQ, fliE*, and *flaG*), were related to the specific clinical outcomes; 3) still-unknown GIs are present within the *H. pylori* genome and putative metabolic islands are linked with the clinical outcomes; 4) *H. pylori* genome carries multiple CRISPRs, albeit without Cas genes detected. Their increased presence is linked to the occurrence of GU. We also found that 5) the “clinical outcome-specific genes” were enriched for functional classes involved in the utilization of inorganic ions in the GC and GU groups, suggesting involvement of the microbial ion transport pathways in driving GC/GU pathological processes.

*H. pylori* infection presents a diagnostic and therapeutic conundrum, since the outcomes and severity of *H. pylori* infection vary significantly among different populations and regions [34]. In this study, we combined the analysis of *H. pylori* genomic population structure with exploration of the associations of four major gastric disorders in Shanghai occurrence with specific *H. pylori* genes colonizing these patients. Regarding the population structure, *H. pylori* gradually undergoes genetic variation and recombination. These gradual changes, combined with historically limited migrations of populations between major continental regions, resulted in the rise of locally restricted strain clusters. In contrast, recent trends associated with increased human mobility and the large-scale international export of food products could potentially influence this population structure. This could be particularly evident in the major cities that serve as international hubs. There are few reports of the population structure of *H. pylori* in China. Here, we provide an analysis of the contemporary *H. pylori*-Shi. Despite Shanghai being one of the major centers of global exchange, the isolates found in this study were overwhelmingly clustered with those in the East Asia population. Although all our samples were collected from the local population, the remarkable cluster stability is consistent with the theory that *H. pylori* colonization of individuals occurs only very early in life and remains consistent with the place of origin of the human migrants [8]. This genomic cluster stability within the East Asia region matched the unique epidemiological landscape with a high local incidence of severe gastric diseases. These findings support the importance of the regional *H. pylori* genomic basis in the development of specific gastric diseases within the local population.

The subsequent analysis of genetic features of *H. pylori* isolates from patients with different gastric diseases aimed to identify the linkage of specific genes to specific clinical outcomes. The goal was to provide clues for future studies to identify the pathological mechanisms of these disorders. Core genes are thought to be related to housekeeping functions and evolutionary stability. These genes have been traditionally thought to be less likely to be linked to the pathogenic properties of the microbes. In contrast, the dispensable genes are related to genome plasticity and evolutionary adaptation to specific conditions in the niche occupied by the organism and thus could be linked to persistence and immune evasion [35]. Presently, the core genome consisted of 1,146 gene clusters, while dispensable genes were the source of genetic variability between the strains and accounted for almost a third of each gene pool. Consistently, we found that most core genes were involved in basic bacterial structure and metabolism. However, there was also a high proportion of functionally unknown genes, which could contain genes functioning in *H. pylori* pathogenesis pathways. As reported, iron deficiency contributed to the risk of GC and iron depletion significantly increased the development of gastric adenocarcinoma in *H. pylori-*infected gerbils [36]. As shown in Figure 3, among the 1,146 core gene clusters, 58 were related to inorganic ion transport and metabolism. Remarkably, as indicated in Table 1, the predominant functional classification of the “outcome-specific” gene clusters in the GC and GU groups was also related to inorganic ion transport and metabolism. Thus, the abundance of specific genes involved in the utilization of inorganic ions in certain *H. pylori* strains could drive colonized-tissue iron depletion and contribute to GC and GU pathogenesis. Moreover, a total of 2,866 genes were detected in the current study. An in-depth analysis of all newly defined genes was beyond the scope of this manuscript. All sequences from this study have been shared in the gene repository for future studies.

In microbial infections, an arsenal of virulence genes is the major determinant of disease severity [37]. The *cag*PAI was reportedly associated with an increased risk of non-cardiac GC or GU disease [38]. Presently, the prevalence of *cag*PAI was high in all four clinical outcome groups. Notably, most *H. pylori* strains carried partial *cag*PAI. It would be interesting to explore if these partial *cag*PAIs are functional, partially functional, or not at all functional. However, we cannot make such a prediction at present, and future studies are needed for specifically addressing this point. The *H. pylori cag*PAI contains approximately 30 putative genes involved in encoding components of a bacterial type IV secretion system (T4SS) [39]. Interestingly, we did not observe a significant difference in the presence of *cagA*, the most studied exotoxin gene of *H. pylori*, in different clinical outcome groups. This suggests that *cagA* might not be linked with the severity of pathogenicity of *H. pylori* or it might work together with other genes to have an impact on disease development. We observed that significantly more strains possessing *cagP* and less *cagY, cag5*, and *cagH* in the GC group. It is likely that these genes, which operate individually or collaboratively with other genetic elements, influence the severity of disease or disease progression. The virulence factor CagP has been implicated in *H. pylori* adherence to gastric epithelial cells, which facilitates the function of T4SS in enhancing pathogenicity [40]. In contrast, the negative association with GC of *cagY, cag5*, and *cagH* might be explained by their inhibition of T4SS function, which might be a bacterial adaptation mechanism to maximize persistent infection [41,42]. These hypothetical scenarios are only based on comparative genomic analysis without negative control. Thus, further studies of mutual interactions between specific components of *H. pylori cag*PAI are needed to understand whether and how they affect the T4SS and gastric mucosa [43].

Our study revealed that, apart from previously known putative virulence factor genes, genes likely to code for microbial fitness appear to be linked with specific diseases, including those involved in metabolic functions, outer membrane structure, motility, and adhesion ability. These capabilities may allow microbes to adapt and survive better in harsh environments or trigger pathological reactions. The bacterial OMPs of *H. pylori* are thought to play an important role in gastric epithelial contact and may influence the severity of gastric inflammation [11,44]. The adhesin BabA can mediate intimate host cell contact and escape from the host immune response to persist in the gastric mucosa [45]. Whether BabA is required for or has a specific role in pathogenicity is unknown. However, we observed the *babA/hopS* gene presence status was associated with GC, suggesting that *babA* might be related to an increased risk of severe gastric disease.

Another major surface structure of *H. pylori* is lipopolysaccharide (LPS). The molecular patterns of LPS are very diverse, enabling *H. pylori* to evade the immune system and alter its interaction with epithelial cells [46]. As reported, the FutA and FutB fucosyltransferases are correlated with certain LPS expression patterns [47]. Presently, a positive association was evident between the presence of genes *futA* and *futB* and GC. The coverage copy numbers of *futA* and *futB* in each strain of each group were also different. The copy numbers were greater in the GC versus GU group, the GU versus CAG group, and the CAG versus CSG group. These findings indicated that the status of *futA* and *futB* might be associated with the pathogenicity of *H. pylori* and could accelerate the progression of disease. However, it should be noted that those genes are phase variable, and even if present, they would be not functional if they are in the off phase.

The motility and adhesion abilities of *H. pylori* conferred by flagellar genes are thought to be required for both successful initial colonization of the gastric mucosa and severe pathological outcomes [48,49]. Our comparative genomic analysis revealed the statistical differences in the presence and copy numbers of several flagellar genes among different clinical outcome groups. While this result may appear counterintuitive, studies have linked the occurrences of flagellar genes with *H. pylori* biofilm formation and stability [12]. Absence of genes responsible for the integrity of flagellar structures and motility might enhance biofilm formation. While considered to be “acutely” less pathogenic than planktonic microbes [50], biofilms might uniquely contribute to genesis of cancer. Moreover, the missing flagellar genes could also result from genome attrition after *H. pylori* has established chronic colonization. In that case, flagella would become an energy burden.

In *H. pylori* isolates, no other GIs were reported, except for *cag*PAI. Here, we found several putative GIs prevalent in *H. pylori* strains. For example, HPGI-1 possessed genes related to aerobic oxidation and ATP synthesis. HPGI-2 contained genes that participate in protein metabolism. Remarkably, HPGI-3 contained genes encoding the Holliday junction resolvase-like protein involved in homologous recombination and chromosome segregation, which was previously found only in eukaryotes including *Schizosaccharomyces pombe* and humans [51]. Our current findings reveal that these genes may also have important biological functions in *H. pylori*. Although several genes in these three predominant GIs found in *H. pylori* could be linked to metabolic adaptability, there are more novel genes with unknown functions that need to be explored.

Despite some implications for virulence in other microbial species [18,52], the structure and function of CRISPRs in *H. pylori* have not been reported. The present study explored the association between CRISPR features and clinical outcomes in *H. pylori* clinical isolates. The finding that significantly more GU isolates possessed CRISPRs suggests that the status of the presence of CRISPRs can be associated with clinical outcomes. It is particularly interesting that this association occurred in the absence of Cas, which may suggest additional enzymatic components that work with CRISPR sequences in *H. pylori*. Furthermore, the occurrence of CRISPRs might be a stress reaction of *H. pylori* toward the harsh environment in the gastric mucosa with certain diseases. The findings provide novel insights into the biological importance of CRISPR and highlight the need for future studies on their role in *H. pylori*.

One limitation of our study is that we could obtain only one isolate from the GC patient. To remedy this, we incorporated 10 reference sequences from GC patients’ *H. pylori* strains and found that they were very similar to our strain in terms of unique gene expression. Thus, future studies are needed to further validate our findings using subsequent GC *H. pylori* isolates from the Shanghai patient population.

In conclusion, the present study systematically explored the genomic features of *H. pylori* and their association with different clinical outcomes. The findings provide further evidence that *H. pylori* possesses unique and currently unknown genetic characteristics. Although the direct impact of our findings on the diagnosis and therapy of *H. pylori* infection cannot be fully realized presently, we believe that our findings will be seminal in informing future studies. These future studies will evaluate the mutual relationship of gastric disorder pathogenesis with the specific parts of the *H. pylori* genome revealed in our study.

## Supplementary Material

Supplemental Material

## References

[1] Crowe SE. Helicobacter pylori Infection. N Engl J Med. 2019;380(12):1158–1165. 30893536 10.1056/NEJMcp1710945

[2] Zamani M, Ebrahimtabar F, Zamani V, et al. Systematic review with meta-analysis: the worldwide prevalence of Helicobacter pylori infection. Aliment Pharmacol Ther. 2018;47(7):868–876. 29430669 10.1111/apt.14561

[3] Robinson K, Atherton JC. The spectrum of Helicobacter mediated diseases. Annu Rev Pathol. 2021;16(1):123–144. 33197219 10.1146/annurev-pathol-032520-024949

[4] Suerbaum S, Josenhans C. Helicobacter pylori evolution and phenotypic diversification in a changing host. Nature Rev Microbiol. 2007;5(6):441–452. 17505524 10.1038/nrmicro1658

[5] Noto JM, Chopra A, Loh JT, et al. Pan-genomic analyses identify key Helicobacter pylori pathogenic loci modified by carcinogenic host microenvironments. Gut. 2018;67(10):1793–1804. 28924022 10.1136/gutjnl-2017-313863 PMC5857411

[6] Muñoz-Ramírez ZY, Mendez-Tenorio A, Kato I, et al. Whole genome sequence and phylogenetic analysis show Helicobacter pylori strains from Latin America Have followed a unique evolution pathway. Front Cell Infect Microbiol. 2017;7:50. 28293542 10.3389/fcimb.2017.00050 PMC5328995

[7] Kumar N, Mariappan V, Baddam R, et al. Comparative genomic analysis of Helicobacter pylori from Malaysia identifies three distinct lineages suggestive of differential evolution. Nucleic Acids Res. 2015;43(1):324–335. 25452339 10.1093/nar/gku1271 PMC4288169

[8] Falush D, Wirth T, Linz B, et al. Traces of human migrations in Helicobacter pylori populations. Science (New York, NY). 2003;299(5612):1582–1585. 10.1126/science.1080857 12624269

[9] Suzuki R, Shiota S, Yamaoka Y. Molecular epidemiology, population genetics, and pathogenic role of Helicobacter pylori. Infect Genet Evol. 2012;12(2):203–213. 22197766 10.1016/j.meegid.2011.12.002 PMC3294018

[10] Nejati S, Karkhah A, Darvish H, et al. Influence of Helicobacter pylori virulence factors CagA and VacA on pathogenesis of gastrointestinal disorders. Microb Pathog. 2018;117:43–48. 29432909 10.1016/j.micpath.2018.02.016

[11] Yamaoka Y, Ojo O, Fujimoto S, et al. Helicobacter pylori outer membrane proteins and gastroduodenal disease. Gut. 2006;55(6):775–781. 16322107 10.1136/gut.2005.083014 PMC1856239

[12] Hathroubi S, Zerebinski J, Ottemann KM. Helicobacter pylori biofilm involves a multigene stress-biased response, including a structural role for flagella. mBio. 2018;9(5). 10.1128/mBio.01973-18 PMC6212823 30377283

[13] Cover TL, Blanke SR. Helicobacter pylori VacA, a paradigm for toxin multifunctionality. Nature Rev Microbiol. 2005;3(4):320–332. 15759043 10.1038/nrmicro1095

[14] Nell S, Estibariz I, Krebes J, et al. Genome and methylome variation in Helicobacter pylori with a cag Pathogenicity Island during early stages of human infection. Gastroenterology. 2018;154(3):612–623.e7. 10.1053/j.gastro.2017.10.014 29066327

[15] Bolinger H, Kathariou S. The current state of macrolide resistance in campylobacter spp.: trends and impacts of resistance mechanisms. Appl Environ Microbiol. 2017;83(12). 10.1128/AEM.00416-17 PMC5452823 28411226

[16] Jang H, Woo J, Lee Y, et al. Draft genomes of Cronobacter sakazakii strains isolated from dried spices bring unique insights into the diversity of plant-associated strains. Stand Genomic Sci. 2018;13(1):35. 30519380 10.1186/s40793-018-0339-6 PMC6267090

[17] Avrani S, Wurtzel O, Sharon I, et al. Genomic island variability facilitates Prochlorococcus-virus coexistence. Nature. 2011;474(7353):604–608. 21720364 10.1038/nature10172

[18] Bikard D, Hatoum-Aslan A, Mucida D, et al. CRISPR interference can prevent natural transformation and virulence acquisition during in vivo bacterial infection. Cell Host Microbe. 2012;12(2):177–186. 22901538 10.1016/j.chom.2012.06.003

[19] Bangpanwimon K, Sottisuporn J, Mittraparp-Arthorn P, et al. CRISPR-like sequences in Helicobacter pylori and application in genotyping. Gut Pathog. 2017;9(1):65. 29177012 10.1186/s13099-017-0215-8 PMC5693588

[20] Maiden MC, Jansen Van Rensburg MJ, Bray JE, et al. MLST revisited: the gene-by-gene approach to bacterial genomics. Nature Rev Microbiol. 2013;11(10):728–736. 23979428 10.1038/nrmicro3093 PMC3980634

[21] Mendoza-Elizalde S, Ac C-M, Zuñiga G, et al. Inference from the analysis of genetic structure of Helicobacter pylori strains isolates from two paediatric patients with recurrent infection. BMC Microbiol. 2019;19(1):184. 31395006 10.1186/s12866-019-1554-z PMC6686460

[22] Jin Y, Deng J, Liang J, et al. Efficient bacteria capture and inactivation by cetyltrimethylammonium bromide modified magnetic nanoparticles. Colloids Surf B Biointerfaces. 2015;136:659–665. 26496472 10.1016/j.colsurfb.2015.10.009

[23] Schubert M, Lindgreen S, Orlando L. AdapterRemoval v2: rapid adapter trimming, identification, and read merging. BMC Res Notes. 2016;9(1):88. 26868221 10.1186/s13104-016-1900-2 PMC4751634

[24] Luo R, Liu B, Xie Y, et al. SOAPdenovo2: an empirically improved memory-efficient short-read de novo assembler. Gigascience. 2012;1(1):18. 23587118 10.1186/2047-217X-1-18 PMC3626529

[25] Coil D, Jospin G, Ae D. A5-miseq: an updated pipeline to assemble microbial genomes from Illumina MiSeq data. Bioinformatics. 2015;31(4):587–589. 25338718 10.1093/bioinformatics/btu661

[26] Powell S, Forslund K, Szklarczyk D, et al. eggNOG v4.0: nested orthology inference across 3686 organisms. Nucleic Acids Res. 2014;42(D1):D231–9. 24297252 10.1093/nar/gkt1253 PMC3964997

[27] Kelley DR, Liu B, Delcher AL, et al. Gene prediction with Glimmer for metagenomic sequences augmented by classification and clustering. Nucleic Acids Res. 2012;40(1):e9. 22102569 10.1093/nar/gkr1067 PMC3245904

[28] Chan PP, Lowe TM. tRNAscan-SE: searching for tRNA Genes in Genomic Sequences. Methods Mol Biol. 2019;1962:1–14. 31020551 10.1007/978-1-4939-9173-0_1 PMC6768409

[29] Lagesen K, Hallin P, Rødland EA, et al. RNAmmer: consistent and rapid annotation of ribosomal RNA genes. Nucleic Acids Res. 2007;35(9):3100–3108. 17452365 10.1093/nar/gkm160 PMC1888812

[30] Sayers S, Li L, Ong E, et al. Victors: a web-based knowledge base of virulence factors in human and animal pathogens. Nucleic Acids Res. 2019;47(D1):D693–d700. 30365026 10.1093/nar/gky999 PMC6324020

[31] Bertelli C, Laird MR, Williams KP, et al. IslandViewer 4: expanded prediction of genomic islands for larger-scale datasets. Nucleic Acids Res. 2017;45(W1):W30–w5. 28472413 10.1093/nar/gkx343 PMC5570257

[32] Cover TL. Helicobacter pylori diversity and gastric cancer risk. mBio. 2016;7(1):e01869–15. 26814181 10.1128/mBio.01869-15 PMC4742704

[33] Ra A, Ls L, Dt M, et al. Genomic-sequence comparison of two unrelated isolates of the human gastric pathogen Helicobacter pylori. Nature. 1999;397(6715):176–180. 9923682 10.1038/16495

[34] Chen Y, Segers S, Blaser MJ. Association between Helicobacter pylori and mortality in the NHANES III study. Gut. 2013;62(9):1262–1269. 23303440 10.1136/gutjnl-2012-303018 PMC3834579

[35] Kumar N, Albert MJ, Al Abkal H, et al. What constitutes an Arabian Helicobacter pylori ? Lessons from comparative genomics. Helicobacter. 2017;22(1):e12323. 10.1111/hel.12323 27277215

[36] Noto JM, Gaddy JA, Lee JY, et al. Iron deficiency accelerates Helicobacter pylori-induced carcinogenesis in rodents and humans. J Clin Invest. 2013;123(1):479–492. 23257361 10.1172/JCI64373 PMC3533289

[37] De Reuse H, Bereswill S. Ten years after the first Helicobacter pylori genome: comparative and functional genomics provide new insights in the variability and adaptability of a persistent pathogen: table 1. FEMS Immunol Med Microbiol. 2007;50(2):165–176. 17567280 10.1111/j.1574-695X.2007.00244.x

[38] Ahmadzadeh A, Ghalehnoei H, Farzi N, et al. Association of CagPAI integrity with severeness of Helicobacter pylori infection in patients with gastritis. Pathol Biol. 2015;63(6):252–257. 26530303 10.1016/j.patbio.2015.09.004

[39] Tegtmeyer N, Wessler S, Backert S. Role of the cag-pathogenicity island encoded type IV secretion system in Helicobacter pylori pathogenesis. Febs J. 2011;278(8):1190–1202. 21352489 10.1111/j.1742-4658.2011.08035.x PMC3070773

[40] Zw Z, Dorrell N, Bw W, et al. Helicobacter pylori adherence to gastric epithelial cells: a role for non-adhesin virulence genes. J Med Microbiol. 2002;51(6):495–502. 12018657 10.1099/0022-1317-51-6-495

[41] Barrozo RM, Hansen LM, Lam AM, et al. CagY is an immune-sensitive regulator of the Helicobacter pylori type IV secretion system. Gastroenterology. 2016;151(6):1164–1175.e3. 10.1053/j.gastro.2016.08.014 PMC5124400 27569724

[42] Saito H, Yamaoka Y, Ishizone S, et al. Roles of virD4 and cagG genes in the cag pathogenicity island of Helicobacter pylori using a Mongolian gerbil model. Gut. 2005;54(5):584–590. 15831899 10.1136/gut.2004.058982 PMC1774503

[43] Salama NR, Hartung ML, Müller A. Life in the human stomach: persistence strategies of the bacterial pathogen Helicobacter pylori. Nature Rev Microbiol. 2013;11(6):385–399. 23652324 10.1038/nrmicro3016 PMC3733401

[44] De Jonge R, Pot RG, Loffeld RJ, et al. The functional status of the Helicobacter pylori sabB adhesin gene as a putative marker for disease outcome. Helicobacter. 2004;9(2):158–164. 15068418 10.1111/j.1083-4389.2004.00213.x

[45] Sheu BS, Sheu SM, Yang HB, et al. Host gastric Lewis expression determines the bacterial density of Helicobacter pylori in babA2 genopositive infection. Gut. 2003;52(7):927–932. 12801945 10.1136/gut.52.7.927 PMC1773709

[46] Maldonado RF, Sá-Correia I, Valvano MA. Lipopolysaccharide modification in Gram-negative bacteria during chronic infection. FEMS Microbiol Rev. 2016;40(4):480–493. 27075488 10.1093/femsre/fuw007 PMC4931227

[47] Nilsson C, Skoglund A, Moran AP, et al. An enzymatic ruler modulates Lewis antigen glycosylation of Helicobacter pylori LPS during persistent infection. Proc Natl Acad Sci U S A. 2006; 103:2863–2868. 10.1073/pnas.0511119103 PMC1413829 16477004

[48] Scott DR, Marcus EA, Wen Y, et al. Gene expression in vivo shows that Helicobacter pylori colonizes an acidic niche on the gastric surface. Proc Natl Acad Sci U S A. 2007; 104:7235–7240. 10.1073/pnas.0702300104 PMC1855417 17438279

[49] Kao CY, Sheu BS, Sheu SM, et al. Higher motility enhances bacterial density and inflammatory response in dyspeptic patients infected with Helicobacter pylori. Helicobacter. 2012;17(6):411–416. 23066970 10.1111/j.1523-5378.2012.00974.x

[50] Blanchette-Cain K, Hinojosa CA, Akula Suresh Babu R, et al. Streptococcus pneumoniae biofilm formation is strain dependent, multifactorial, and associated with reduced invasiveness and immunoreactivity during colonization. mBio. 2013;4(5):e00745–13. 24129258 10.1128/mBio.00745-13 PMC3812715

[51] Ip SC, Rass U, Blanco MG, et al. Identification of Holliday junction resolvases from humans and yeast. Nature. 2008;456(7220):357–361. 19020614 10.1038/nature07470

[52] Louwen R, Staals RH, Endtz HP, et al. van der Oost J. The role of CRISPR-Cas systems in virulence of pathogenic bacteria. Microbiol Mol Biol Rev. 2014;78(1):74–88. 24600041 10.1128/MMBR.00039-13 PMC3957734

