# Supplementary material for: Genomic population structure of Helicobacter pylori Shanghai isolates and identification of genomic features uniquely linked with pathogenicity
Source: Virulence. 2021 Apr 27;12(1):1258–70. doi: 10.1080/21505594.2021.1920762 (PMC8081043; doi:10.1080/21505594.2021.1920762)
Supplement: Supplemental Material [file KVIR_A_1920762_SM7170.zip › downloadFromZipFile.pdf]

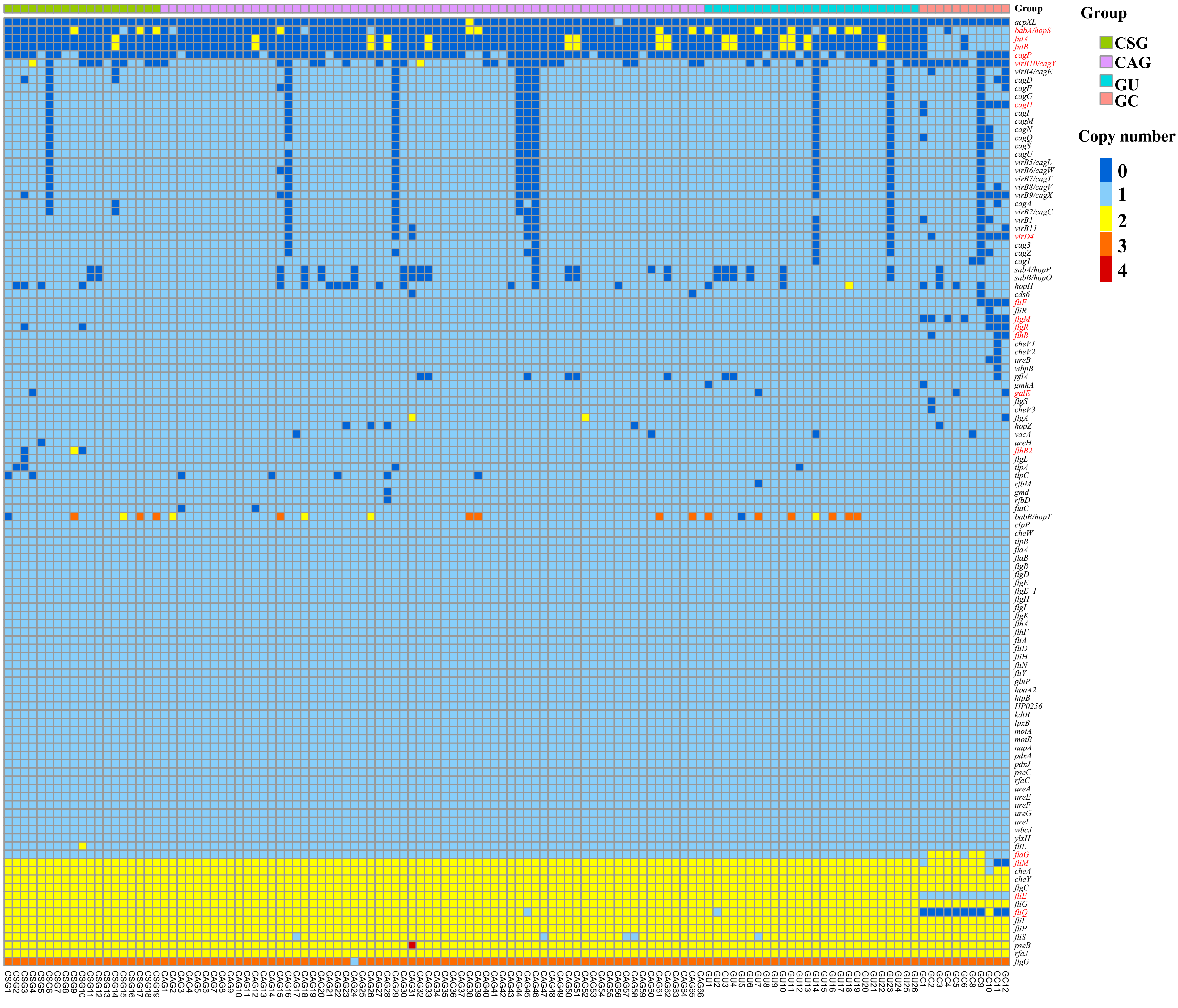
Group
CSG
CAG
GU
GC
Copy number
0
1
2
3
4
acpXL
babA/hopS
futA
futB
cagP
virB10/cagY
virB4/cagE
cagD
cagF
cagG
cagH
cagI
cagM
cagN
cagQ
cagS
cagU
virB5/cagL
virB6/cagW
virB7/cagT
virB8/cagV
virB9/cagX
cagA
virB2/cagC
virB1
virB11
virD4
cag3
cagZ
cag1
sabA/hopP
sabB/hopO
hopH
cds6
fliF
fliR
flgM
flgR
flhB
cheV1
cheV2
ureB
wbpB
pflA
gmhA
galE
flgS
cheV3
flgA
hopZ
vacA
ureH
flhB2
flgL
tlpA
tlpC
rfbM
gmd
rfbD
futC
babB/hopT
clpP
cheW
tlpB
flaA
flaB
flgB
flgD
flgE
flgE_1
flgH
flgI
flgK
flhA
flhF
fliA
fliD
fliH
fliN
fliY
gluP
hpaA2
htpB
HP0256
kdtB
lpxB
motA
motB
napA
pdxA
pdxJ
pseC
rfaC
ureA
ureE
ureF
ureG
ureI
wbcJ
ylxH
fliL
flaG
fliM
cheA
cheY
flgC
fliE
fliG
fliQ
fliI
fliP
fliS
pseB
rfaJ
flgG

Supplemental Figure 1. *H. pylori* strains from patients with major clinical outcomes demonstrate outcome-specific variation in gene copy numbers among the 115 virulence-associated genes

Heat map of the copy numbers of the 115 virulence-associated genes in each isolate shows different genes organized along the Y-axis and individual strains arranged along the X axis according to the clinical outcome groups marked with different colors in the first row. Different colors in each grid represent the copy numbers of certain gene as shown in the right icon.
