# Supplementary material for: Genomic population structure of Helicobacter pylori Shanghai isolates and identification of genomic features uniquely linked with pathogenicity
Source: Virulence. 2021 Apr 27;12(1):1258–70. doi: 10.1080/21505594.2021.1920762 (PMC8081043; doi:10.1080/21505594.2021.1920762)
Supplement: Supplemental Material [file KVIR_A_1920762_SM7170.zip › table.pdf]

Supplemental Table 1. The demographic information of patients in the study

Groups	Number of patients (%)
**Sex**	
**Male**	61 (54.5%)
**Female**	51 (45.5%)
**Age group**	
**19-29 years of age**	8 (7.1%)
**30-49 years of age**	35 (31.3%)
**50-69 years of age**	56 (50.0%)
**≥70 years of age**	13 (11.6%)
**Pathological diagnosis**	
**Chronic Superficial Gastritis**	19 (17.0%)
**Chronic atrophic gastritis**	66 (58.9%)
**Peptic ulcer**	26 (23.2%)
**Gastric cancer**	1 (0.9%%)
**Geographical origin**	
**Shanghai, China**	112 (100.0%)

Supplemental Table 2. The detailed information of the 10 reference strains

Number	Name	Accession number	Geographic origin
**GC2**	CPY6081	NZ_AKNN00000000	Japan
**GC3**	CPY6261	NZ_AKNO00000000	Japan
**GC4**	CPY6271	NZ_AKNP00000000	Japan
**GC5**	CPY6311	NZ_AKNQ00000000	Japan
**GC6**	HLJ039	NZ_JAAA00000000	China
**GC7**	Hp238	NZ_CP010013	China
**GC8**	29CaP	NZ_CP012907	Mexico
**GC9**	HPML1	AP014710	China
**GC10**	HPML2	AP014711	China
**GC11**	HPML3	AP014712	China

Supplemental Table 3. The information of the 120 comparative *H. pylori* sequences used in population structure analysis

Populations	Number of sequences used
**EastAsia**	
**EAsia**	30
**Amerind**	10
**Maori**	10
**Asia2**	10
**Africa1**	
**WAfrica**	10
**SAfrica**	10
**Africa2**	10
**NEAfrica**	10
**Europe**	10
**Sahul**	10

All these sequences were exported from the PubMLST database without other original genome or background information.

Supplemental Table 4. General genomic features and the genome accessions of the 112 *H. pylori*-Shi isolates

Clinical diagnosis	names	Length	Contigs	N50-1	Scaffolds	N50-2	GC%	rRNA	tRNA	Genome accessions
**Chronic Superficial Gastritis**										
	CSG1	1575816	22	164151	22	164151	38.77	2	36	JAEPYH000000000
	CSG2	1581069	34	173357	25	173891	38.70	2	36	JAEPYG000000000
	CSG3	1599709	108	45221	35	84373	38.61	2	36	JAEPYF000000000
	CSG4	1579575	35	105408	28	121189	38.68	2	36	JAEPYE000000000
	CSG5	1589094	33	144551	27	144551	38.65	2	36	JAEPYD000000000
	CSG6	1544169	26	174341	25	174341	38.86	2	36	JAEPYC000000000
	CSG7	1604125	21	167116	21	167116	38.72	2	36	JAEPYB000000000
	CSG8	1595400	27	105037	27	105037	38.66	2	36	JAEPYA000000000
	CSG9	1570566	25	164762	24	169194	38.82	2	36	JAEPXZ000000000
	CSG10	1645544	57	65630	53	80440	38.58	2	36	JAEPXY000000000
	CSG11	1605774	39	105553	37	120837	38.62	2	36	JAEPXX000000000
	CSG12	1610778	30	143652	29	164967	38.64	2	36	JAEPXW000000000
	CSG13	1601054	21	160933	20	164249	38.69	2	36	JAEPXV000000000
	CSG14	1575751	21	130111	18	161930	38.78	2	36	JAEPXU000000000
	CSG15	1551793	21	159239	20	159239	38.82	2	36	JAEPXT000000000
	CSG16	1568578	23	164705	21	164705	38.78	2	36	JAEPXS000000000
	CSG17	1625183	37	146766	36	160733	38.65	2	36	JAEPXR000000000
	CSG18	1603819	23	157069	22	166790	38.72	2	36	JAEPXQ000000000
	CSG19	1622418	16	215037	14	215037	38.68	2	36	JAEPXP000000000
**Chronic atrophic gastritis**										
	CAG1	1603463	22	166124	22	166124	38.72	2	36	JAEQAV000000000
	CAG2	1593200	31	137858	30	137858	38.67	2	36	JAEQAU000000000
	CAG3	1610969	22	165189	22	165189	38.70	2	36	JAEQAT000000000

CAG4	1604125	21	167116	21	167116	38.72	2	36	JAEQAS000000000
CAG5	1603513	22	167117	22	167117	38.72	2	36	JAEQAR000000000
CAG6	1603857	22	166566	22	166566	38.72	2	36	JAEQAQ000000000
CAG7	1603675	21	166563	21	166563	38.72	0	36	JAEQAP000000000
CAG8	1603904	22	167115	22	167115	38.72	2	36	JAEQAO000000000
CAG9	1603738	21	166566	21	166566	38.72	2	36	JAEQAN000000000
CAG10	1604433	21	167117	21	167117	38.72	2	36	JAEQAM000000000
CAG11	1651146	28	117315	28	117315	38.50	2	36	JAEQAL000000000
CAG12	1601900	19	187335	19	187335	38.67	2	36	JAEQAK000000000
CAG13	1572581	30	98380	30	98380	38.79	2	36	JAEQAJ000000000
CAG14	1606534	24	166368	24	166368	38.67	2	36	JAEQAI000000000
CAG15	1546749	22	216511	21	216511	38.91	2	36	JAEQAH000000000
CAG16	1528788	41	78465	33	80754	38.90	2	36	JAEQAG000000000
CAG17	1555512	33	98758	28	105946	38.85	2	36	JAEQAF000000000
CAG18	1611059	24	164618	21	164618	38.70	1	36	JAEQAE000000000
CAG19	1571909	37	106065	29	191940	38.67	2	36	JAEQAD000000000
CAG20	1625739	31	158411	26	203528	38.66	2	36	JAEQAC000000000
CAG21	1595825	30	144261	25	144261	38.68	2	36	JAEQAB000000000
CAG22	1588145	43	109280	33	167404	38.64	1	36	JAEQAA000000000
CAG23	1587486	43	82658	28	161100	38.73	0	36	JAEPZZ000000000
CAG24	1582323	24	164640	24	164640	38.77	2	36	JAEPZY000000000
CAG25	1536509	18	186096	16	186096	38.92	2	36	JAEPZX000000000
CAG26	1639241	37	100503	28	183564	38.67	2	36	JAEPZW000000000
CAG27	1576248	27	89480	21	124915	38.72	1	36	JAEPZV000000000
CAG28	1609087	37	114844	28	116475	38.65	2	36	JAEPZU000000000
CAG29	1566325	31	73551	28	110804	38.76	2	36	JAEPZT000000000
CAG30	1619955	37	117416	34	117416	38.66	2	36	JAEPZS000000000
CAG31	1692034	89	57353	87	59067	38.63	2	36	JAEPZR000000000
CAG32	1638336	45	77865	37	81462	38.82	1	36	JAEPZQ000000000
CAG33	1653713	32	121511	32	121511	38.81	2	36	JAEPZP000000000
CAG34	1604289	21	167118	21	167118	38.72	2	36	JAEPZO000000000
CAG35	1603736	21	166565	21	166565	38.72	2	36	JAEPZN000000000
CAG36	1603679	21	166563	21	166563	38.72	2	36	JAEPZM000000000
CAG37	1607143	24	167115	24	167115	38.71	2	36	JAEPZL000000000
CAG38	1609349	24	166339	24	166339	38.69	2	36	JAEPZK000000000
CAG39	1583529	21	220594	21	220594	38.75	2	36	JAEPZJ000000000
CAG40	1655133	31	131929	30	159453	38.52	2	36	JAEPZI000000000
CAG41	1568355	27	111399	24	166088	38.81	2	36	JAEPZH000000000
CAG42	1565267	18	178437	17	178437	38.81	2	36	JAEPZG000000000
CAG43	1614105	38	75961	36	77123	38.60	2	36	JAEPZF000000000
CAG44	1605541	28	77084	27	93799	38.63	2	36	JAEPZE000000000
CAG45	1582217	33	108695	30	108695	38.73	2	36	JAEPZD000000000
CAG46	1569344	25	106080	24	107533	38.73	2	36	JAEPZC000000000
CAG47	1567362	24	125457	21	167519	38.81	2	36	JAEPZB000000000
CAG48	1629908	29	107023	27	113404	38.64	2	36	JAEPZA000000000
CAG49	1573810	31	81076	29	84682	38.80	2	36	JAEPYZ000000000
CAG50	1652999	32	121511	32	121511	38.81	2	36	JAEPYY000000000
CAG51	1653388	32	121511	32	121511	38.81	2	36	JAEPYX000000000
CAG52	1596667	51	105912	51	105912	38.74	2	36	JAEPYW000000000
CAG53	1607898	24	174370	24	174370	38.65	2	36	JAEPYV000000000
CAG54	1626737	52	164518	51	164518	38.77	2	36	JAEPYU000000000
CAG55	1551660	27	131887	25	164651	38.87	2	36	JAEPYT000000000
CAG56	1610753	31	108428	31	108428	38.69	2	36	JAEPYS000000000
CAG57	1610753	29	99121	27	160856	38.76	2	36	JAEPYR000000000
CAG58	1617359	30	132441	28	159665	38.62	2	36	JAEPYQ000000000
CAG59	1586413	20	165012	20	165012	38.68	2	36	JAEPYP000000000
CAG60	1582581	20	167576	20	167576	38.71	2	36	JAEPYO000000000
CAG61	1563838	16	173265	16	173265	38.90	2	36	JAEPYN000000000
CAG62	1652961	33	121511	32	121511	38.81	2	36	JAEPYM000000000
CAG63	1562751	28	122163	26	164979	38.84	2	36	JAEPYL000000000
CAG64	1610031	25	200092	24	200092	38.67	2	36	JAEPYK000000000

	CAG65	1560217	26	125418	23	167273	38.86	2	36	JAEPYJ000000000
	CAG66	1547428	30	154907	26	154907	38.88	2	36	JAEPYI000000000
**Peptic ulcer**										
	GU1	1557232	32	88446	27	88446	38.78	2	36	JAEPXO000000000
	GU2	1604691	26	204169	24	204169	38.68	2	36	JAEPXN000000000
	GU3	1654268	33	121511	33	121511	38.81	2	36	JAEPXM000000000
	GU4	1656130	35	121505	35	121505	38.81	2	36	JAEPXL000000000
	GU5	1632863	31	104640	30	104640	38.57	2	36	JAEPXK000000000
	GU6	1659615	30	127837	30	127837	38.47	2	36	JAEPXJ000000000
	GU7	1606183	21	218880	20	218880	38.70	2	36	JAEPXI000000000
	GU8	1631461	33	111551	29	119533	38.55	2	36	JAEPXH000000000
	GU9	1639482	21	165282	20	165282	38.60	2	36	JAEPXG000000000
	GU10	1605178	15	176268	14	184326	38.71	2	36	JAEPXF000000000
	GU11	1633538	18	164639	17	164639	38.63	2	36	JAEPXE000000000
	GU12	1618290	25	130655	22	167194	38.61	2	36	JAEPXD000000000
	GU13	1592744	20	169279	20	169279	38.70	2	36	JAEPXC000000000
	GU14	1531743	30	160883	29	160883	38.92	2	36	JAEPXB000000000
	GU15	1576529	36	69490	35	79897	38.78	2	36	JAEPXA000000000
	GU16	1574829	20	214481	19	214481	38.81	2	36	JAEPWZ000000000
	GU17	1652468	29	105176	28	105176	38.52	2	36	JAEPWY000000000
	GU18	1607000	17	181892	16	223656	38.70	2	36	JAEPWX000000000
	GU19	1610990	28	83508	28	83508	38.61	2	36	JAEPWW000000000
	GU20	1593980	24	159229	23	165819	38.71	2	36	JAEPWV000000000
	GU21	1603877	23	157069	22	166791	38.72	2	36	JAEPWU000000000
	GU22	1602633	22	163515	21	163515	38.68	2	36	JAEPWT000000000
	GU23	1523641	23	131223	21	159881	38.93	2	36	JAEPWS000000000
	GU24	1580618	28	160705	28	160705	38.79	2	36	JAEPWR000000000
	GU25	1574321	29	107786	27	107786	38.79	2	36	JAEPWQ000000000
	GU26	1566147	29	82729	28	84582	38.91	2	36	JAEPWP000000000
**Gastric cancer**	GC1	1618222	45	103341	38	103341	38.66	0	36	JAEPWO000000000

Abbreviations: N50, arrange all contigs or scaffolds according to length from long to short, add their sequences in this order and when the added length reaches 50% of the total length, the length of the final sequence is N50.

Supplemental Table 5. The ENOG (evolutionary Non-supervised Orthologous Groups) numbers of the 208 function unknown genes detected by COG database

ENOG4108KB7	ENOG4108KB7	ENOG4108KB7	ENOG4108KB7	ENOG4105RRP	ENOG4105RRP	ENOG4107QWG	ENOG4108E4B
ENOG4108E4B	ENOG4108Z4T	ENOG4108UHV	ENOG4106AD4	ENOG41081ZF	ENOG4107XHT	ENOG4105E4Y	ENOG4105F4G
ENOG4106NCB	ENOG41073XF	ENOG4105KIS	ENOG410821Z	ENOG4106E51	ENOG4108SDW	ENOG4105D3Y	ENOG4108KBJ
ENOG4105DPE	ENOG4105DAW	ENOG41062BV	ENOG4105DIJ	ENOG4105QJI	ENOG4108UXA	ENOG4105CQP	ENOG410679F
ENOG4108JQ6	ENOG4105DIN	ENOG4108S9E	ENOG4105P1V	ENOG4105CSD	ENOG4105FPI	ENOG4107Y58	ENOG4105CHR
ENOG4108RUE	ENOG4107RN3	ENOG410833R	ENOG4108W5G	ENOG41084FT	ENOG4106T8Z	ENOG4105K9M	ENOG4105GEZ
ENOG4105DCY	ENOG4105CKI	ENOG4108X8F	ENOG4105F3Y	ENOG41066ST	ENOG4105HFZ	ENOG4106AKH	ENOG4107MTB
ENOG4106VXV	ENOG410640C	ENOG4108V0Z	ENOG4109060	ENOG41080V7	ENOG4105NNI	ENOG4106AKH	ENOG4108YYC
ENOG4107ZDD	ENOG410819M	ENOG4105SR9	ENOG4108QDQ	ENOG4105PS2	ENOG410886E	ENOG4108ZUB	ENOG4105CUR
ENOG4108YXA	ENOG4105ZY0	ENOG4105GF5	ENOG4105P1V	ENOG4105QDU	ENOG410674Z	ENOG41082Y0	ENOG4105MRU
ENOG4106JE1	ENOG4105YDS	ENOG4105ZFS	ENOG410638D	ENOG4106A4J	ENOG4106FE7	ENOG4108IUZ	ENOG4107YD3
ENOG4105CQP	ENOG4105I9H	ENOG41086QS	ENOG410609H	ENOG41090B2	ENOG4108ETT	ENOG4105FD2	ENOG41068HQ
ENOG4105C1H	ENOG4105ER4	ENOG4105E63	ENOG4105WHM	ENOG4105KHW	ENOG4108N0T	ENOG41082BX	ENOG4105Y57

ENOG4107V0X	ENOG4107Z0M	ENOG4105ZHV	ENOG4105MU7	ENOG410679F	ENOG4106BRS	ENOG4107YSJ	ENOG4107I6C
ENOG4108A2D	ENOG4105QTX	ENOG4107ZW7	ENOG4105P3W	ENOG4108730	ENOG4106EXC	ENOG4105T1H	ENOG4107T1F
ENOG4108QRP	ENOG4105VMA	ENOG4105GAX	ENOG4106962	ENOG4105DIC	ENOG4106EPT	ENOG4107ZDD	ENOG41064HH
ENOG4105D4I	ENOG4105IM2	ENOG41075W0	ENOG4107D3I	ENOG4108UGZ	ENOG4105Y7S	ENOG4107R5Z	ENOG4105X75
ENOG4108P97	ENOG4105KB8	ENOG4105WGM	ENOG4105K7Z	ENOG4107YBA	ENOG4105C5H	ENOG4107MMI	ENOG4108KA0
ENOG41079X0	ENOG410613S	ENOG4105DCY	ENOG4107YVB	ENOG4107YV6	ENOG410621X	ENOG4105NF4	ENOG4105K7D
ENOG4105XZ2	ENOG4105PNC	ENOG4105PRZ	ENOG41060B0	ENOG410696D	ENOG4106B5A	ENOG41069EC	ENOG41069BQ
ENOG410800X	ENOG4105ZB8	ENOG4108W9V	ENOG4105JDQ	ENOG4107BJB	ENOG4108Y7S	ENOG4107A0K	ENOG4106UJ4
ENOG4106XI8	ENOG4105YKR	ENOG4108R4X	ENOG4108UMD	ENOG4108X98	ENOG4108VBP	ENOG4106Y2E	ENOG4106ZQ9
ENOG4105C7N	ENOG4106C92	ENOG4108TS0	ENOG4108266	ENOG41079KP	ENOG4105VFU	ENOG4108F8H	ENOG41061E7
ENOG4108X2Q	ENOG4106APR	ENOG41061KU	ENOG4105K6Z	ENOG4105IFD	ENOG4105EI9	ENOG4105JZY	ENOG4105DV6
ENOG4107F67	ENOG4105XIT	ENOG4108A47	ENOG4105T34	ENOG4105H3B	ENOG4108QGE	ENOG4105KHU	ENOG41067YZ
ENOG4108S5E	ENOG41060R5	ENOG4107XJD	ENOG41076M9	ENOG410695P	ENOG4108XUR	ENOG4105TIY	ENOG4106Y3B

The information of the function unknown genes could be accessed using the corresponding ENOG numbers in COG database (http://eggnogdb.embl.de/#/app/home/).

Supplemental Table 6. Virulence genes detected from the genomes of 112 *H. pylori*-Shi and 10 reference strains against the VFDB database

Genes	Number and % gene positive isolates in each clinical category				*P*
	CAG(n=66)	CSG(n=19)	GU(n=26)	GC(n=11)	
cag1	65(98.5%)	19(100.0%)	25(96.2%)	9(81.8%)	0.069
cag3	64(97.0%)	19(100.0%)	24(92.3%)	10(90.9%)	0.268
cagA	62(93.9%)	17(89.5%)	25(96.2%)	9(81.8%)	0.357
cagD	61(92.4%)	16(84.2%)	24(92.3%)	8(72.7%)	0.180
cagF	60(90.9%)	18(94.7%)	24(92.3%)	9(81.8%)	0.673
cagG	61(92.4%)	18(94.7%)	24(92.3%)	10(90.9%)	1.000
cagH	61(92.4%)	18(94.7%)	24(92.3%)	6(54.5%)	0.008
cagI	61(92.4%)	18(94.7%)	24(92.3%)	9(81.8%)	0.634
cagM	61(92.4%)	18(94.7%)	24(92.3%)	10(90.9%)	1.000
cagN	61(92.4%)	18(94.7%)	24(92.3%)	9(81.8%)	0.634
cagP	9(13.6%)	3(15.8%)	4(15.4%)	6(54.5%)	0.027
cagQ	61(92.4%)	18(94.7%)	24(92.3%)	8(72.7%)	0.208
cagS	62(93.9%)	18(94.7%)	24(92.3%)	9(81.8%)	0.523
cagU	61(92.4%)	18(94.7%)	24(92.3%)	10(90.9%)	1.000
cagZ	62(93.9%)	19(100.0%)	24(92.3%)	9(81.8%)	0.252
virB1	62(93.9%)	19(100.0%)	24(92.3%)	8(72.7%)	0.075
virB2/cagC	61(92.4%)	17(89.5%)	25(96.2%)	10(90.9%)	0.751
virB4/cagE	61(92.4%)	17(89.5%)	24(92.3%)	8(72.7%)	0.227

virB5/cagL	61(92.4%)	18(94.7%)	24(92.3%)	10(90.9%)	1.000
virB6/cagW	60(90.9%)	18(94.7%)	24(92.3%)	10(90.9%)	1.000
virB7/cagT	61(92.4%)	18(94.7%)	24(92.3%)	10(90.9%)	1.000
virB8/cagV	61(92.4%)	18(94.7%)	24(92.3%)	9(81.8%)	0.634
virB9/cagX	60(90.9%)	17(89.5%)	24(92.3%)	7(63.6%)	0.092
virB10/cagY	44(66.7%)	11(57.9%)	17(65.4%)	1(9.1%)	0.004
virB11	61(92.4%)	19(100.0%)	24(92.3%)	9(81.8%)	0.315
virD4/cag5	61(92.4%)	19(100.0%)	24(92.3%)	6(54.5%)	0.003
flaA	66(100.0%)	19(100.0%)	26(100.0%)	11(100.0%)	\
flaB	66(100.0%)	19(100.0%)	26(100.0%)	11(100.0%)	\
flaG	66(100.0%)	19(100.0%)	26(100.0%)	11(100.0%)	\
flgA	66(100.0%)	19(100.0%)	26(100.0%)	10(90.9%)	0.090
flgB	66(100.0%)	19(100.0%)	26(100.0%)	11(100.0%)	\
flgC	66(100.0%)	19(100.0%)	26(100.0%)	11(100.0%)	\
flgD	66(100.0%)	19(100.0%)	26(100.0%)	11(100.0%)	\
flgE	66(100.0%)	19(100.0%)	26(100.0%)	11(100.0%)	\
flgE1	66(100.0%)	19(100.0%)	26(100.0%)	11(100.0%)	\
flgG	66(100.0%)	19(100.0%)	26(100.0%)	11(100.0%)	\
flgH	66(100.0%)	19(100.0%)	26(100.0%)	11(100.0%)	\
flgI	66(100.0%)	19(100.0%)	26(100.0%)	11(100.0%)	\
flgK	66(100.0%)	19(100.0%)	26(100.0%)	11(100.0%)	\
flgL	66(100.0%)	18(94.7%)	26(100.0%)	11(100.0%)	\
flgM	66(100.0%)	19(100.0%)	26(100.0%)	4(36.4%)	0.000
flgR	66(100.0%)	17(89.5%)	26(100.0%)	8(72.7%)	0.000
flgS	66(100.0%)	19(100.0%)	26(100.0%)	10(90.9%)	0.090
flhA	66(100.0%)	19(100.0%)	26(100.0%)	11(100.0%)	\
flhB	66(100.0%)	19(100.0%)	26(100.0%)	8(72.7%)	0.001
flhB2	66(100.0%)	17(89.5%)	26(100.0%)	11(100.0%)	0.031
flhF	66(100.0%)	19(100.0%)	26(100.0%)	11(100.0%)	\
fliA	66(100.0%)	19(100.0%)	26(100.0%)	11(100.0%)	\
fliD	66(100.0%)	19(100.0%)	26(100.0%)	11(100.0%)	\
fliE	66(100.0%)	19(100.0%)	26(100.0%)	11(100.0%)	\
fliF	66(100.0%)	19(100.0%)	26(100.0%)	7(63.6%)	0.000
fliG	66(100.0%)	19(100.0%)	26(100.0%)	11(100.0%)	\
fliH	66(100.0%)	19(100.0%)	26(100.0%)	11(100.0%)	\
fliI	66(100.0%)	19(100.0%)	26(100.0%)	11(100.0%)	\
fliL	66(100.0%)	19(100.0%)	26(100.0%)	11(100.0%)	\
fliM	66(100.0%)	19(100.0%)	26(100.0%)	9(81.8%)	0.007
fliN	66(100.0%)	19(100.0%)	26(100.0%)	11(100.0%)	\
fliP	66(100.0%)	19(100.0%)	26(100.0%)	11(100.0%)	\

fliQ	66(100.0%)	19(100.0%)	26(100.0%)	1(9.1%)	0.000
fliR	66(100.0%)	19(100.0%)	26(100.0%)	10(90.9%)	0.090
fliS	66(100.0%)	19(100.0%)	26(100.0%)	11(100.0%)	\
fliY	66(100.0%)	19(100.0%)	26(100.0%)	11(100.0%)	\
babA/hopS	8(12.1%)	4(21.1%)	7(26.9%)	9(81.8%)	0.000
babB/hopT	66(100.0%)	18(94.7%)	25(96.2%)	11(100.0%)	0.209
sabA/hopP	53(80.3%)	17(89.5%)	20(76.9%)	10(90.9%)	0.661
sabB/hopO	54(81.8%)	17(89.5%)	20(76.9%)	10(90.9%)	0.708
hopH	55(83.3%)	15(78.9%)	24(92.3%)	7(63.6%)	0.190
hopZ	62(93.9%)	19(100.0%)	26(100.0%)	10(90.9%)	0.344
cds6	64(97.0%)	19(100.0%)	26(100.0%)	10(90.9%)	0.408
cheA	66(100.0%)	19(100.0%)	26(100.0%)	11(100.0%)	\
acpXL	2(3.0%)	0(0.0%)	0(0.0%)	0(0.0%)	\
cheV1	66(100.0%)	19(100.0%)	26(100.0%)	10(90.9%)	0.090
cheV2	66(100.0%)	19(100.0%)	26(100.0%)	10(90.9%)	0.090
cheV3	66(100.0%)	19(100.0%)	26(100.0%)	10(90.9%)	0.090
cheW	66(100.0%)	19(100.0%)	26(100.0%)	11(100.0%)	\
cheY	66(100.0%)	19(100.0%)	26(100.0%)	11(100.0%)	\
clpP	66(100.0%)	19(100.0%)	26(100.0%)	11(100.0%)	\
futA	8(12.1%)	1(5.3%)	6(23.1%)	9(81.8%)	0.000
futB	8(12.1%)	1(5.3%)	6(23.1%)	9(81.8%)	0.000
futC	64(97.0%)	19(100.0%)	26(100.0%)	11(100.0%)	1.000
galE	66(100.0%)	18(94.7%)	25(96.2%)	9(81.8%)	0.015
gluP	66(100.0%)	19(100.0%)	26(100.0%)	11(100.0%)	\
gmd	65(98.5%)	19(100.0%)	26(100.0%)	11(100.0%)	1.000
gmhA	66(100.0%)	19(100.0%)	25(96.2%)	10(90.9%)	0.098
HP0256	66(100.0%)	19(100.0%)	26(100.0%)	11(100.0%)	\
hpaA2	66(100.0%)	19(100.0%)	26(100.0%)	11(100.0%)	\
htpB	66(100.0%)	19(100.0%)	26(100.0%)	11(100.0%)	\
kdtB	66(100.0%)	19(100.0%)	26(100.0%)	11(100.0%)	\
lpxB	66(100.0%)	19(100.0%)	26(100.0%)	11(100.0%)	\
motA	66(100.0%)	19(100.0%)	26(100.0%)	11(100.0%)	\
motB	66(100.0%)	19(100.0%)	26(100.0%)	11(100.0%)	\
napA	66(100.0%)	19(100.0%)	26(100.0%)	11(100.0%)	\
pdxA	66(100.0%)	19(100.0%)	26(100.0%)	11(100.0%)	\
pdxJ	66(100.0%)	19(100.0%)	26(100.0%)	11(100.0%)	\
pflA	60(90.9%)	19(100.0%)	24(92.3%)	10(90.9%)	0.618
pseB	66(100.0%)	19(100.0%)	26(100.0%)	11(100.0%)	\
pseC	66(100.0%)	19(100.0%)	26(100.0%)	11(100.0%)	\
rfaC	66(100.0%)	19(100.0%)	26(100.0%)	11(100.0%)	\

rfaJ	66(100.0%)	19(100.0%)	26(100.0%)	11(100.0%)	\
rfbD	65(98.5%)	19(100.0%)	26(100.0%)	11(100.0%)	1.000
rfbM	66(100.0%)	19(100.0%)	25(96.2%)	11(100.0%)	0.459
tlpA	65(98.5%)	17(89.5%)	25(96.2%)	11(100.0%)	0.176
tlpB	66(100.0%)	19(100.0%)	26(100.0%)	11(100.0%)	\
tlpC	61(92.4%)	17(89.5%)	26(100.0%)	11(100.0%)	0.373
ureA	66(100.0%)	19(100.0%)	26(100.0%)	11(100.0%)	\
ureB	66(100.0%)	19(100.0%)	26(100.0%)	9(81.8%)	0.007
ureE	66(100.0%)	19(100.0%)	26(100.0%)	11(100.0%)	\
ureF	66(100.0%)	19(100.0%)	26(100.0%)	11(100.0%)	\
ureG	66(100.0%)	19(100.0%)	26(100.0%)	11(100.0%)	\
ureH	66(100.0%)	18(94.7%)	26(100.0%)	11(100.0%)	0.246
ureI	66(100.0%)	19(100.0%)	26(100.0%)	11(100.0%)	\
wbcJ	66(100.0%)	19(100.0%)	26(100.0%)	11(100.0%)	\
wbpB	66(100.0%)	19(100.0%)	26(100.0%)	10(90.9%)	0.090
ylxH	66(100.0%)	19(100.0%)	26(100.0%)	11(100.0%)	\
vacA	66(100.0%)	19(100.0%)	26(100.0%)	11(100.0%)	\

All genes detected in 112 *H. pylori*-Shi and 10 reference strains are searched against the VFDB database and 115 virulence-associated genes are found. The presence of these genes in strains of four clinical outcome groups is analyzed and shown in this table.

Supplemental Table 7. The 20 other virulence-associated genes detected by Victors database

Genes	Number and % gene positive isolates in each clinical category				*P*
	CAG (n=66)	CSG (n=19)	GU (n=26)	GC (n=11)	
gltS	66 (100.0%)	19 (100.0%)	26 (100.0%)	11 (100.0%)	\
groEL	66 (100.0%)	19 (100.0%)	26 (100.0%)	11 (100.0%)	\
putP	66 (100.0%)	19 (100.0%)	26 (100.0%)	11 (100.0%)	\
ureC	66 (100.0%)	19 (100.0%)	26 (100.0%)	11 (100.0%)	\
hspR	66 (100.0%)	19 (100.0%)	26 (100.0%)	11 (100.0%)	\
msbA	66 (100.0%)	19 (100.0%)	26 (100.0%)	11 (100.0%)	\
omp8	66 (100.0%)	19 (100.0%)	26 (100.0%)	11 (100.0%)	\
hemC	66 (100.0%)	19 (100.0%)	26 (100.0%)	11 (100.0%)	\
prtC	66 (100.0%)	19 (100.0%)	26 (100.0%)	11 (100.0%)	\
horB	66 (100.0%)	19 (100.0%)	26 (100.0%)	11 (100.0%)	\
rfaD	66 (100.0%)	19 (100.0%)	26 (100.0%)	11 (100.0%)	\
trbB	66 (100.0%)	19 (100.0%)	26 (100.0%)	11 (100.0%)	\
trpB	66 (100.0%)	19 (100.0%)	26 (100.0%)	11 (100.0%)	\
tufA	66 (100.0%)	19 (100.0%)	26 (100.0%)	11 (100.0%)	\
hetA	66 (100.0%)	19 (100.0%)	26 (100.0%)	11 (100.0%)	\
galU	66 (100.0%)	19 (100.0%)	26 (100.0%)	11 (100.0%)	\
serA	66 (100.0%)	19 (100.0%)	26 (100.0%)	11 (100.0%)	\
alpA	66 (100.0%)	19 (100.0%)	26 (100.0%)	11 (100.0%)	\
katA	17 (25.8%)	2 (10.5%)	3 (11.5%)	1 (9.1%)	0.263
homB	60 (90.9%)	19 (100.0%)	24 (92.3%)	11 (100.0%)	0.605

Supplemental Table 8. The distribution of putative GIs among four clinical outcome groups

GIs	Groups				*P*
	CSG (n=19)	CAG (n=66)	GU (n=26)	GC (n=11)	
**Number detected (%)**	19 (100%)	65 (98.5%)	26 (100%)	10 (90.9%)	-
**Average GI loads**	3.1	2.7	2.8	2.3	0.3897

The putative GIs sequences were commonly found among all strains from all patient groups. Note that the average loads are shared by all the strains in each group, including the strains with a complete absence of GI.

Supplemental Table 9. The distribution of CRISPRs among four clinical outcome groups

CRISPRs	Group				*P*
	CSG (n=19)	CAG (n=66)	GU (n=26)	GC (n=11)	
**Number detected (%)**	8 (42.1%)	32 (48.5%)	21 (80.8%)	5 (45.5%)	0.021

Significant difference in CRISPIR frequencies (*P*=0.021) is detected among the clinical outcomes with in particular linked with the occurrence of GU.

Supplemental Table 10. Eighty-eight STs detected from the 112 *H. pylori*-Shi isolates

	CSG	CAG	GU	GC
**ST3274**	2	12	1	-
**ST3327**	-	5	2	-
**ST3315**	2	-	-	-
**ST3326**	-	2	-	-
**ST3334**	1	1	-	-
**ST3345**	-	1	1	-
	CSG	CAG	GU	GC
**ST3292**	-	1	-	-
**ST3296**	-	1	-	-
**ST3299**	-	1	-	-
**ST3300**	-	1	-	-
**ST3301**	-	1	-	-
**ST3302**	-	1	-	-
**ST3303**	-	1	-	-
**ST3304**	-	1	-	-
**ST3305**	-	1	-	-
**ST3306**	-	1	-	-
**ST3307**	-	1	-	-
**ST3308**	-	1	-	-
**ST3309**	-	1	-	-
**ST3311**	-	1	-	-
**ST3312**	-	1	-	-
**ST3316**	-	1	-	-
**ST3317**	-	1	-	-
**ST3319**	-	1	-	-
**ST3320**	-	1	-	-
**ST3324**	-	1	-	-
**ST3325**	-	1	-	-
**ST3329**	-	1	-	-
**ST3330**	-	1	-	-

**ST3333**	-	1	-	-
**ST3335**	-	1	-	-
**ST3337**	-	1	-	-
**ST3338**	-	1	-	-
**ST3339**	-	1	-	-
**ST3340**	-	1	-	-
**ST3342**	-	1	-	-
**ST3344**	-	1	-	-
**ST3353**	-	1	-	-
**ST3356**	-	1	-	-
**ST3358**	-	1	-	-
**ST3360**	-	1	-	-
**ST3365**	-	1	-	-
**ST3366**	-	1	-	-
**ST3368**	-	1	-	-
**ST3369**	-	1	-	-
**ST3372**	-	1	-	-
**ST3373**	-	1	-	-
**ST3375**	-	1	-	-
**ST3378**	-	1	-	-
**ST3380**	-	1	-	-
**ST3381**	-	1	-	-

	CSG	CAG	GU	GC
**ST3279**	1	-	-	-
**ST3313**	1	-	-	-
**ST3321**	1	-	-	-
**ST3322**	1	-	-	-
**ST3323**	1	-	-	-
**ST3328**	1	-	-	-
**ST3346**	1	-	-	-
**ST3348**	1	-	-	-
**ST3352**	1	-	-	-
**ST3354**	1	-	-	-
**ST3361**	1	-	-	-
**ST3362**	1	-	-	-
**ST3363**	1	-	-	-
**ST3376**	1	-	-	-
**ST3314**	-	-	1	-
**ST3318**	-	-	1	-
**ST3331**	-	-	1	-
**ST3332**	-	-	1	-
**ST3336**	-	-	1	-
**ST3341**	-	-	1	-
**ST3343**	-	-	1	-
**ST3347**	-	-	1	-
**ST3349**	-	-	1	-

**ST3350**	-	-	1	-
**ST3351**	-	-	1	-
**ST3355**	-	-	1	-
**ST3357**	-	-	1	-
**ST3359**	-	-	1	-
**ST3364**	-	-	1	-
**ST3367**	-	-	1	-
**ST3370**	-	-	1	-
**ST3371**	-	-	1	-
**ST3374**	-	-	1	-
**ST3377**	-	-	1	-
**ST3379**	-	-	1	-
**ST3382**	-	-	1	-
**ST3310**	-	-	-	1
